# Supplementary material for: Disease risk analysis in sea turtles: A baseline study to inform conservation efforts
Source: PLoS One. 2020 Oct 23;15(10):e0230760. doi: 10.1371/journal.pone.0230760 (PMC7584443; doi:10.1371/journal.pone.0230760)
Supplement: S1 File — (DOCX) [file pone.0230760.s001.docx]

# APPENDICES

Table of Contents

[APPENDICES 1](#_Toc51521678)

[S1. Mock Clutch Translocation 2](#_Toc51521679)

[1.1. Problem description 2](#_Toc51521680)

[1.2. Risk communication 2](#_Toc51521681)

[1.3. Hazard identification 2](#_Toc51521682)

[1.4. Risk assessment 4](#_Toc51521683)

[1.5. Risk management 6](#_Toc51521684)

[1.6. Implementation 7](#_Toc51521685)

[S2. Handbook for Sea Turtle Disease Risk Analysis (risk assessment workshop) 8](#_Toc51521686)

[S3. The handbook for management workshop 14](#_Toc51521687)

[S4. Bacteria 18](#_Toc51521688)

[4.1. Gram negative bacteria 18](#_Toc51521689)

[4.2. Gram positive bacteria 27](#_Toc51521690)

[4.3. Not defined by gram staining 31](#_Toc51521691)

[4.4. Mixed bacterial infections 33](#_Toc51521692)

[S5. Fungi 35](#_Toc51521693)

[S6. Parasites 43](#_Toc51521694)

[S7. Viruses 59](#_Toc51521695)

[S8. Non-infectious diseases of sea turtles 64](#_Toc51521696)

[References 72](#_Toc51521697)

## S1. Mock Clutch Translocation

### 1.1. Problem description

A clear problem description that is not influenced by political and social issues would help the efficiency of DRA [1]. The scope is determined through precise information such as scientific name, exact locations, and number of animals to be translocated and the frequency of such actions. The goal of the risk analysis should be stated **clearly** [2]. For example, for clutch translocation to identify and assess the likelihood of the hazard(s) being introduced and spreading or becoming established in (the area of translocation)’.

*Information to assist in identifying hazards, assessing risks and exploring options to manage risk can be found in the current study*.

### 1.2. Risk communication

A risk communication strategy should be developed for two purposes: 1) identifying interested parties (stakeholders and experts); 2) defining the timing and the means of communication with them [2].

### 1.3. Hazard identification

The hazards likely to be associated with the species under consideration should be identified. The hazard identification process is not as exhaustive as explained in Appendices S4-8. Only putative disease-causing-hazards are considered that relate to the population, the region and the scenario under consideration. An example for clutch translocation is shown in following table, this table can vary for different translocation scenarios, regions and species of sea turtles. Table 1 is derived from appendices S4-8, see these sections for reference(s).

Table 1. Potential disease hazards for sea turtle egg clutch translocation

| **Infectious Hazards** | | | |
| --- | --- | --- | --- |
| **Fungi** | **Parasites** | **Gram Negative Bacteria** | **Gram Positive Bacteria** |
| *Allescheria spp.*  *(Pseud)allescheria boydii*  *Absidia*  *Cephalosporium*  *curtipes var.*  *uredinicola*  *Cladosporium sp*  *Chrysosporium*  *Cunninghamella*  *Cylindrocarpon*  *Emericella*  *Fusarium solani*  *Homodendrum*  *Saksenaea vasiformis*  *Scedosporium*  *aurantiacum*  *Thielavia* | *Diplotesticulata, Oligochaeta*  ***Coleoptera***  *Elater spp., Lanelater sallei, Omorgus suberosus, Pimelia sp.*  ***Diptera***  *Sarcophaga (Parasarcophaga)*  *crassipalpis, Sarcotachina subcylindric*  ***Orthoptera***  *Gryllotalpidae (Scapteriscus didactylus)* | *Citrobacter freundii*  *Citrobacter youngae*  *Enterobacter cloacae*  *Morganella*  *morganii*  *Proteus penneri*  *Proteus vulgaris*  *Pseudomonas spp.*  *Serratia odorifera*  *Vibrio mimicus* | *Bacillus spp.*  *Enterococcus spp.* |
| Non-infectious hazards | | | |
| Terrestrial predators  Poaching  Marine predators | | | |

Considering the hazard table (Table 1) the following questions should be answered to decide if a risk assessment is required. **There should be sufficient capability and confidence to rule out the presence of pathogens, or to claim that they are not hazardous** [2].

*“3.2 Is the live animal or germplasm under consideration a potential vehicle for the pathogenic agent?*

*3.3 Is the pathogenic agent present in the area from which the animals or germplasm are sourced?*

*3.4 Are there zones from which the animals or germplasm will be sourced that are free of the pathogenic agent?*

*3.5 Is the pathogenic agent already present in the area to which animals or germplasm are to be translocated and which will be affected by the planned activity?”* (Page 47, [2]).

### 1.4. Risk assessment

Risk assessment should be conducted for each hazard and the populations of interest. For example, for clutch translocation, potentially susceptible species may be terrestrial and aquatic predators as well as humans if the hazard has zoonotic potential. High risk pathogens should be selected. The criteria by which the hazard is selected depends on the translocation situation and can be done through expert workshops, paired rankings or scenario trees. Table 2 is an example of risk assessment using paired ranking and some pathogens such as Coleoptera (beetles) may not be an issue in some regions. The health risk ranking should be done through expert ranking and the risk can vary from negligible to minimal, moderate or high.

Table 2. The health risk of pathogen for translocated eggs

| Pathogen | Health risk | Evaluation. |
| --- | --- | --- |
| **Fungi** | High  Or Moderate  Or Minimal  Or Negligible |  |
| *Fusarium solani* |  | Widely distributed, associated with mass mortalities in relocated nests. Zoonotic. |
| *Cladosporium sp.* |  | Hatching failure is reported; not enough data. |
| **Parasites** |  |  |
| Coleoptera |  | Different species of beetles are found in nests. |
| Diptera |  | Dead-decayed eggs were reported to be attacked by flies. |
| **Bacteria** |  |  |
| Enterobacter |  | Widely spread component of gastrointestinal flora; zoonotic. |
| *Citrobacter freundii* |  | A confirmed contaminant of eggs. Poses risk in translocation to the handler and local wildlife. |

A scenario tree can also be drawn to identify the various biological (risk) pathways leading to the translocated animals and also susceptible animals or humans being exposed to translocated animals. Figure 1 shows an example of a scenario tree for clutch translocation. In this scenario tree, release assessment was also considered to be able to estimate the impact on the environment and ecosystem. When the assessment is done the critical control points should be defined and ranked with high or medium priority (red triangles in Figure 1).


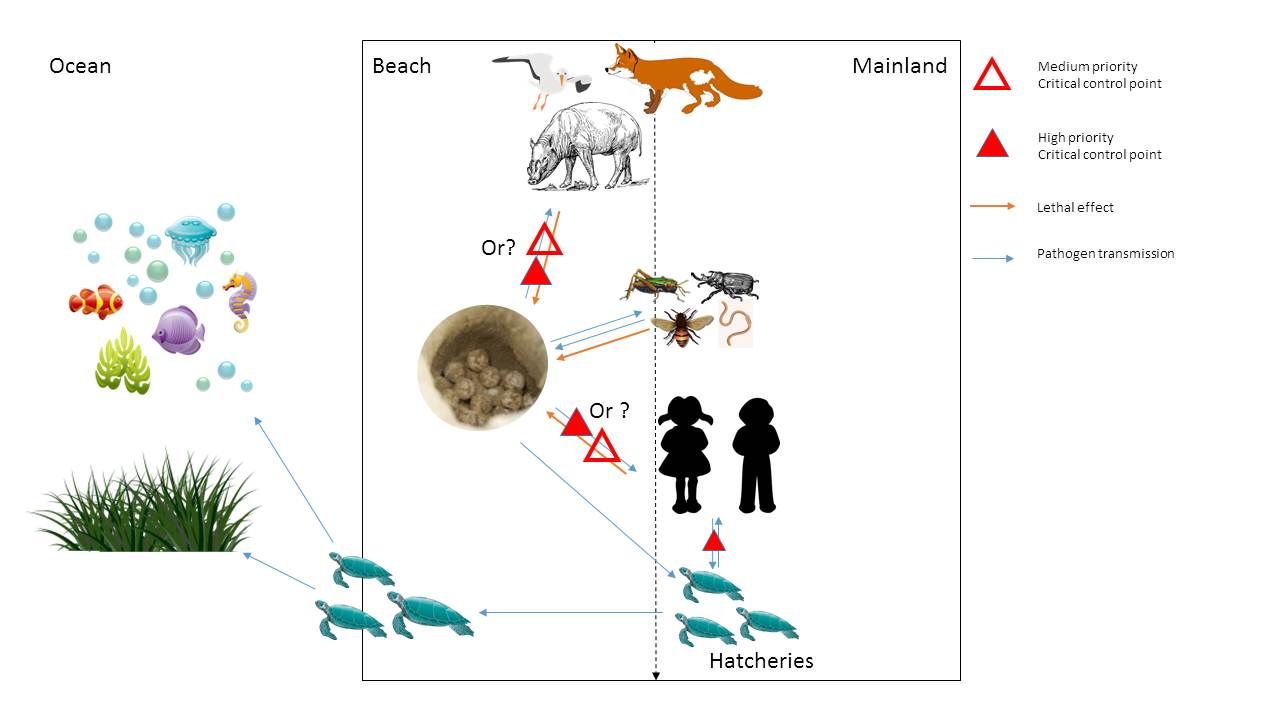


Figure 1. The clutch translocation scenario, pathogen transmission pathways, lethal effects of predators and critical control points (The vectors, characters and icons in this figure were downloaded from the public domain <https://pixabay.com/> and were modified according to its licence).

The results or conclusions from each “*risk assessment*” procedure should be summarised and provided for risk management (Risk estimation).

### 1.5. Risk management

*Risk evaluation*: If the risk estimate is greater than the acceptable level for stakeholders, options for mitigating the risk should be evaluated.

*Option evaluation and selection*: An objective will be formulated that clearly defines the risk mitigations and is technically, operationally and economically feasible and ideally is based on scientific principles and a risk analysis [2].

### 1.6. Implementation

The final decision should be made to implement the risk mitigation measure(s). The overall procedure needs to be followed up by monitoring and review. In some cases, if the risk is high and there is no applicable measures to mitigate the risk the translocation may be abandoned.

## S2. Handbook for Sea Turtle Disease Risk Analysis (risk assessment workshop)

The workshop’s objective is to prioritise the importance of infectious and non-infectious diseases for conservation, surveillance and research.

**Step 1: Forming small groups for discussions (5 minutes)**

You will work in small groups to carry out a sea turtle disease risk analysis (DRA) following the process outlined in this workbook.

You will have about 5 minutes to become acquainted with your group members and their contributions to sea turtle health.

**Step 2: Hazard identification (15 minutes)**

During this step of any health risk assessment you would normally use books, journals and many more resources to gather information about infectious and non-infectious diseases that threaten the animals’ survival.

*For this workshop this information is being provided for you.*

*Note: The list provided here is based on current shared knowledge and there are many more bacterial, viral, fungal and parasitic infections, present and influencing disease in sea turtles. The exact epidemiology and clinical manifestation of many of the pathogens that are known to affect sea turtles are not thoroughly understood. The same applies to non-infectious diseases where the aetiology and the influence on the population may not be completely studied.*

To make the process easier, you will see:

- Non-infectious diseases are described in one table.
- The pathogens have been categorised in four different tables of bacteria, fungi, parasites and viruses.
- **Each group will only evaluate one category.**

Read the information carefully about sea turtle health hazards (Material 1: Pathogen tables and health problems table).

**Step 3: Hazard ranking (60 minutes)**

We will work on each part and will have a discussion after this step.

Considering the time and budget restraints to do a full health assessment on all of these hazards, if you must choose only three hazards that represent the highest potential risk to sea turtle health, which three would you select? Fill the table below and write why your group has chosen these three hazards.

| Hazards | Reasoning |
| --- | --- |
|  |  |
|  |  |
|  |  |
|  |  |
|  |  |

**Step 4: One Health (30 minutes)**

A successful DRA should consider the study population in the context of the environment. Disease exposure not only affects a population but also the habitat, humans and companion animals. “*One Health*” considers the inter-dependent health of the environment, humans and animals.

Use *Material 1: Pathogen table* to evaluate the risk associated with physical contact with sea turtles. This can be from a human's perspective; for example, the risk of transferring infections through meat and egg consumption. The other aspect is the risk of pathogen transmission from humans to sea turtles; for example, handling sea turtles in rehabilitation centres or during nest relocation (or “clutch translocation”) activities.

We will work on this in our groups and will have a discussion after this step.

*If you are working on pathogen tables answer questions 1 to 4 and if you are working on non-infectious health problems answer question 5 and 6:*

1. What is the main zoonotic pathogen of concern in your group?

2. Are there any pathogens being transferred from human to sea turtles?

3. What is the main problematic pathogen in captivity?

4. Are there any pathogens to be considered as a risk for aquaculture?

5. What is your insight about the cultural dimensions of interacting with sea turtles?

6. What is the socio-economic advantage of sea turtle conservation?

**Step 5: General discussion about sea turtle health (10 minutes)**

Now that we have finished reviewing sea turtle health hazards we can start a general conversation about putting conservation in practice. In other words, how can we use this information to help conservation?

## S3. The handbook for management workshop

Introduction to group members.

Introduction to DRA presented by Narges Mashkour.

**Part one: Reviewing the risk assessment outcomes from previous workshops**

| **Infectious health hazards** | | |
| --- | --- | --- |
| **Parasite** | | *Spirorchiidae* |
|  |  | Annelids |
|  |  | Arthropods |
| **Virus** | | Herpesvirus in association with FP |
|  |  | Papillomavirus |
| **Bacteria** | | *Enterobacteriaceae* and antibiotic resistant bacteria |
|  |  | *Strep iniae, salmonella typhimurium,* Ecoli |
|  |  | *Pseudomonas spp.* Klebsiella |
| **Fungi** | | *Fusarium solani* |
|  |  | *Penicillium spp.* |
|  |  | *Cladosporium spp.* |
| **Non-infectious health hazards** | | |
|  | Anthropogenic (Plastic, by-catch, boat strike) | |
|  | Environmental | |
|  | Medical | |

**Part two: Defining management options**

In this section “management options” will be proposed and for each option the level of “effectiveness” and “feasibility” is ranked **from 1 being the least feasible/effective to 10 the most feasible/effective**. Desirably, based on these criteria a decision about that option is made.

Non-infectious Health hazards: Macroplastic

| **Management Option** | **Effectiveness** | **Feasibility** | **Decision** |
| --- | --- | --- | --- |
|  |  |  |  |
|  |  |  |  |
|  |  |  |  |
|  |  |  |  |
|  |  |  |  |
|  |  |  |  |
|  |  |  |  |
|  |  |  |  |

Infectious Health Hazard: *Enterobacteriaceae* and antibiotic resistant bacteria

| **Management Option** | **Effectiveness** | **Feasibility** | **Decision** |
| --- | --- | --- | --- |
|  |  |  |  |
|  |  |  |  |
|  |  |  |  |
|  |  |  |  |
|  |  |  |  |
|  |  |  |  |
|  |  |  |  |
|  |  |  |  |
|  |  |  |  |
|  |  |  |  |

**Part three: Critical control points for a mock translocation scenario and diagram**

Consider a scenario for translocating a clutch of egg from Raine Island to mainland. Discuss the high and medium priority critical control points.

## S4. Bacteria

### 4.1. Gram negative bacteria

| Disease hazard | Region reported | Presence in sea turtles | | Outcome of infection (lesion, clinical sign and/or disease)  Symptom in individuals;  Ease of spread, rate of spread;  Diagnostic test or treatment, if available | Zoonotic/transmissible to companion animals | Correlation with climatic/anthropogenic events | Key reference(s) |
| --- | --- | --- | --- | --- | --- | --- | --- |
|  |  | **Captive populations** | **Wild populations** |  |  |  |  |
| *Achromobacter spp.and Achromobacter spanius* | Canary Islands, Spain; Sugözü Beaches, Turkey |  | Loggerhead (*Caretta caretta*), Green (*Chelonia mydas*) | Opportunistic pathogens Heterophilic scleritis  Found in sands in green turtle nests | Can be found in aquatic species |  | [3, 4] |
| *Acinetobacter anittratus* | Florida, USA | * | Green (*Chelonia mydas*) with and without FP | Opportunistic and able to infect tissues damaged by trauma  ulcerative dermatitis and rhinitis and stomatitis, shell disease, bronchopneumonia in captivity |  | Anthropogenic effects can cause trauma and trigger infection | [5, 6] |
| *Acinetobacter lwoffi* | Hawaii, USA |  | Green (*Chelonia mydas*) with and without FP | A low frequency isolation in the survey |  |  | [6] |
| *Acinetobacter calcoaceticus* | Northern Territory, Australia | Loggerhead (*Caretta caretta*) | Green (*Chelonia mydas)*, Hawksbill (*Eretmochelys imbricata*), Loggerhead (*Caretta caretta*) | Oral lesions and conjunctivitis  ulcerative stomatitis, obstructive rhinitis-pneumonia;  Detection: clinical signs and microbial culture of caseous lesions | Potentially zoonotic;  Evidences of conjunctivitis in humans |  | [7, 8] |
| *Aeromonas hydrophilia* | Australia; Hawaii, USA; Spain | Green (*Chelonia mydas*), Loggerhead (*Caretta caretta*) | Green (*Chelonia mydas*), Hawksbill (*Eretmochelys imbricata*), Leatherback (*Dermochelys coriacea*), Loggerhead (*Caretta caretta*) | Traumatic ulcerative skin lesions (align with *Vibrio alginolyticus* and *Staphylococcus* *spp*.);  Ulcerative stomatitis, obstructive rhinitis-pneumonia complex and FP;  9 cases of salt gland infection and mortality in Loggerhead (*Caretta caretta*) along with *Staphylococcus* *spp*., and *Vibrio*  *alginolyticus* [9];  Detection: clinical signs and microbial culture of caseous lesions | Potentially zoonotic | Boat strike, by catching fishing line | [7-11] |
| *Aeromonas sorbia* | Hawaii, USA |  | Green (*Chelonia mydas*) | Found in blood samples, no pathogenicity determined;  Low frequency isolation in the survey |  |  | [10] |
| *Aeromonas popoffii* | Hawaii, USA |  | Green (*Chelonia mydas*) | Found in blood samples, no pathogenicity determined;  Low frequency isolation in the survey |  |  | [10] |
| *Aeromonas caviae* | Hawaii, USA |  | Green (*Chelonia mydas*) | Found in blood samples, no pathogenicity determined;  Low frequency isolation in the survey |  |  | [10] |
| *Alcaligenes faecalis* | Jekyll Island, Georgia, USA; Sugözü Beaches, Turkey |  | Loggerhead (*Caretta caretta*), Green (*Chelonia mydas*) | High frequent in failed hatching;  Egg contents and sand in nest |  |  | [4, 12] |
| *Alcanivorax dieselolei* | Sugözü Beaches, Turkey |  | Green (*Chelonia mydas*) | Found in egg contents |  |  | [4] |
| *Aureobacterium spp.* | Thailand | Green (*Chelonia mydas*), Hawksbill (*Eretmochelys imbricata)* | Green (*Chelonia mydas*) | Isolated from oral cavity and liver in captivity;  Can be from normal skin flora without pathogenicity |  |  | [13] |
| *Burkholderia spp. And B. cepacia* | Canary Islands, Spain; Hawaii, USA |  | Loggerhead (*Caretta caretta*) | Heterophilic blepharitis, heterophilic stomatitis and obstructive rhinitis;  Low frequency isolation |  |  | [3, 10] |
| *Citrobacter spp.* | Florida, USA; Oman Sea and Persian Gulf | * | Black (*Chelonia mydas agassizii*) | Opportunistic and able to infect tissues damaged by trauma  ulcerative dermatitis and rhinitis and stomatitis, shell disease, bronchopneumonia in captivity;  Egg contaminant |  | Anthropogenic effects can cause trauma and trigger infection | [5, 14] |
| *Citrobacter diversus* | Hawaii, USA |  | Green (*Chelonia mydas*) | Isolated in 1 sample out of 22 along with *E. coli* |  |  | [6] |
| *Citrobacter freundii* | Central Mediterranean Sea; East Caribbean Sea; Florida, USA | * | Green (*Chelonia mydas*) with and without FP,  Leatherback (*Dermochelys coriacea*),  Loggerhead (*Caretta caretta*), Olive ridley (*Lepidochelys olivacea)* | Opportunistic pathogen. may correlate to hatching failure;  Cutaneous ulceration which can be septicemic, sloughing skin;  Resistant to ampicillin and Shows 17% to 100% resistance to antibiotic tested | May infect several species;  May pose risk to egg consumers |  | [5, 11, 15-18] |
| *Citrobacter youngae* | Tuscany, Italy | Green (*Chelonia mydas*) | Leatherback (*Dermochelys coriacea*) | May correlate with hatching failure;  Resistant to ampicillin and amoxicillin with clavulanic acid |  |  | [19, 20] |
| *Citrobacter brakii* | Central Mediterranean Sea |  | Loggerhead (*Caretta caretta*) | A low frequent isolation with *Citrobacter freundii* |  |  | [16] |
| *Ochrobactrum anthropi* | Sugözü Beaches, Turkey |  | Green (*Chelonia mydas*) | Found in nest sands | Is an pportunistic human pathogens  Is found in polluted soil |  | [4] |
| *Sphingobacterium multivorum* | Sugözü Beaches, Turkey |  | Green (*Chelonia mydas*) | Found in egg contents | Is a human pathogen  Helps in degradation of  petroleum |  | [4] |
| *Zobellella denitrificans* | Sugözü Beaches, Turkey |  | Green (*Chelonia mydas*) | Isolated in nest and sands | Growth  promoters in agriculture and aquaculture |  | [4] |
| *Enterobacteriacae* | | | | | | | |
| *Edwardsiella spp.* | Northwestern Mexico; Oman Sea and Persian Gulf; Thailand | Green (*Chelonia mydas*), Hawksbill (*Eretmochelys imbricata*) | Black (*Chelonia mydas agassizii*), Green (*Chelonia mydas*) | Potentially pathogenic and opportunistic in sick individuals;  Can be from normal skin flora without pathogenicity;  Collected in oviductal samples in nesting beaches;  *Edwardsiella tarta* is resistant to Streptomycin |  |  | [15, 21] |
| *Enterobacter aerogenes* | Hawaii, USA |  | Green (*Chelonia mydas*) with and without FP, Leatherback (*Dermochelys coriacea*) | Found in samples;  No pathogenicity determined |  |  | [6] |
| *Enterobacter agglomerans* | Hawaii, USA |  | Green (*Chelonia mydas*) | Found in nasal and cloacal samples;  No pathogenicity determined |  |  | [6] |
| *Enterobacter cloacae* | East Caribbean Sea | Leatherback (*Dermochelys coriacea*) | Green (*Chelonia mydas*) with and without FP,  Leatherback (*Dermochelys coriacea*), Loggerhead (*Caretta caretta*) | Opportunistic pathogen – may correlate to hatching failure;  Resistant to ampicillin and Amoxicillin with clavulanic acid | May pose risk to egg consumers |  | [20] |
| *Escherichia coli* | Central Mediterranean Sea; Oman Sea and Persian Gulf | Green (*Chelonia mydas*) | Green (*Chelonia mydas*), Leatherback (*Dermochelys coriacea*), Loggerhead (*Caretta caretta*) | Coliforms;  Resistant to Tetracycline | Zoonotic;  May infect several species | High temperature may increase *E. coli* growth | [3, 7, 16, 22] |
| *Hafnia alvei* | Florida, USA; Hawaii, USA |  | Green (*Chelonia mydas*) with FP | Has been isolated from clinical specimens of animals but the information about pathogenicity is not enough | Commensals of terrestrial reptiles  may also infect fish;  May pose risk to humans |  | [5, 6] |
| *Klebsiella oxytoca* | Florida, USA |  | Green (*Chelonia mydas*) with FP | Pathogenicity not determined;  May be from normal flora without pathogenicity |  |  | [5, 11] |
| *Klebsiella pneumoniae* | Rio de Janeiro, Brazil; USA | Green (*Chelonia mydas)*, Loggerhead (*Caretta caretta*) | Green (*Chelonia mydas)*, Loggerhead (*Caretta caretta*) | Correlated with lower hatching success in natural and relocated nests of Loggerhead (*Caretta caretta*) along with other *Enterobacteriaceae;*    Associated with ulcerative stomatitis in captive hatchling and juvenile Green (*Chelonia mydas*) and Loggerhead (*Caretta caretta*) | Opportunistic and may infect several species |  | [23, 24] |
| *Morganella*  *morganii* | East Caribbean Sea; Oman Sea and Persian Gulf | Leatherback (*Dermochelys coriacea*) | Green (*Chelonia mydas*) | Opportunistic pathogens;  May correlate to hatching failure;  Resistant to ampicillin and Amoxicillin with clavulanic acid | May pose risk to egg consumers |  | [19] |
| *Plesiomonas* | Northwestern Mexico |  | Black (*Chelonia mydas agassizii*) | Potentially pathogenic and opportunistic in sick individuals |  |  | [25] |
| *Providencia (Proteus) spp.* | Canary Islands; Oman Sea; Persian Gulf; Florida, USA |  | Black (*Chelonia mydas agassizii*), Green (*Chelonia mydas*) | May be from normal skin flora without pathogenicity: Costa Rica;  Most frequently isolated from lesions in Canary Island and indicated as one of the causes of the diseases and mortality;  May be egg contaminant;  Maybe resistant to ampicillin, streptomycin and tetracycline | Many reptile species | May be due to habitat pollution | [3, 5, 21] |
| *Proteus mirabilis* | Costa Rica;  Ionian Sea; Sicilian Channel, Italy; South Tyrrhenian Sea |  | Green (*Chelonia mydas*), Loggerhead (*Caretta caretta*) | May be from normal skin flora without pathogenicity: Costa Rica |  |  | [17, 24] |
| *Proteus penneri* | Georgia, USA |  | Loggerhead (*Caretta caretta*) | Hatching failure |  |  | [12] |
| *Proteus vulgaris* | Canary Islands, Spain; Costa Rica; Ionian Sea; Oman Sea and Persian Gulf; Sicilian Channel, Italy; South Tyrrhenian Sea |  | Green (*Chelonia mydas*) without FP, Loggerhead (*Caretta caretta*) | Indicated as non-pathogenic constituent of microflora in costa Rica;  Reported to relate with Loggerhead (*Caretta caretta*) hatching failure in Jekyll Island, Georgia, USA;  Shows 17% to 100% resistance to antibiotic |  | May be due to pollution in the habitat | [12, 17, 21, 24] |
| *Providencia rettgeri* | Ionian Sea; Sicilian Channel, Italy; South Tyrrhenian Sea |  | Loggerhead (*Caretta caretta*) | Shows 62.5% to 94.1% resistance to antibiotic: carbenicillin, cephalothin, oxytetracycline and amoxicillin |  |  | [17] |
| *Shigella* | Oman Sea; Persian Gulf |  | Green (*Chelonia mydas*) | Collected in oviductal samples no pathogenicity was  resistant to ampicillin and maybe tetracycline |  |  | [21] |
| *Flavobacterium spp.* | Northern Australia | Green (*Chelonia mydas*), Hawksbill (*Eretmochelys imbricata*), Loggerhead (*Caretta caretta*) | Green (*Chelonia mydas*), Hawksbill (*Eretmochelys imbricata*), Loggerhead (*Caretta caretta*) | Ulcerative stomatitis, obstructive rhinitis-pneumonia complex and FP;  The frequent bacteria in keratoconjunctivitis, ulcerative blepharitis, Salt- secreting gland infection, Peritonitis along with *Pseudomonas* *spp*.;  Detection: clinical signs and microbial culture of caseous lesions | Potentially zoonotic |  | [7, 8] |
| *Leptospira interrogans* | Baja California, Mexico |  | Green (*Chelonia mydas*) | Leptospirosis;  Detection: culture | Zoonotic;  May infect several species – individuals may act as reservoirs  of some serotypes |  | [26] |
| *Moraxella spp.* | Western Australia, Australia |  | Green (*Chelonia mydas*) | Isolated from liver, lung and kidney along with mycotic infections | Opportunistic environmental pathogens |  | [15] |
| *Pasteurella* | Canary Islands, Spain |  | Green (*Chelonia mydas*),  Leatherback (*Dermochelys coriacea*), Loggerhead (*Caretta caretta*) | Pneumonia, gastrointestinal opportunistic;  In mixed infections causing catarrhal, fibrinous, necropurulent and necrotising enteritis;  Egg contaminant | Potentially zoonotic;  May infect several species |  | [27] |
| *Photobacterium damselae (*subspecies *damselae)* | Tasmania, Australia; New Zealand; Tuscany, Italy |  | Green (*Chelonia mydas*) with and without FP, Leatherback (*Dermochelys coriacea*),  Loggerhead (*Caretta caretta*) | Ulcers and haemorrhagic septicaemia, congested lung showing nodules in parenchyma, blood in body cavity;  Pathogenicity not determined. May be from normal flora without pathogenicity | Sharks, dolphins and shrimps, wild and cultivated fish |  | [20, 28] |
| *Pseudomonas spp.* | Australia; Central Mediterranean Sea; East Caribbean Sea; Hawaii, USA | * | Green (*Chelonia mydas*) with FP, Hawksbill (*Eretmochelys imbricata*), Leatherback (*Dermochelys coriacea*),  Loggerhead (*Caretta caretta*) | Opportunistic pathogen. may correlate to hatching failure;  Ulcerative stomitis and dermatitis along with vibrio alginolyticus;  The frequent bacteria in keratoconjunctivitis, ulcerative blepharitis, Salt- secreting gland infection, Peritonitis along with *Flavobacterium* *spp*.;  Resistant to ampicillin, amoxicillin with clavulanic acid and sulfamethoxazole-trimethoprim | Potentially zoonotic;  May pose risk to egg consumers | Infections in the salt glands due to the removal of foreign material | [6, 7, 19] |
| *Pseudomonas putrefaciens* | Hawaii, USA |  | Green (*Chelonia mydas*) with and without FP | Pathogenicity not determined. May be from normal flora without pathogenicity;  Detection: clinical signs and microbial culture of caseous lesions | Second most frequent bacteria in Aguirre 1994 |  | [6] |
| *Pseudomonas putida* | Hawaii, USA | Green (*Chelonia mydas*) | Green (*Chelonia mydas*) with and without FP | A frequent isolation in the survey (21/32) |  |  | [6] |
| *Pseudomonas fluorescens* | Hawaii, USA | Green (*Chelonia mydas*), Hawksbill (*Eretmochelys imbricata)*, Loggerhead (*Caretta caretta*) | Green (*Chelonia mydas*)  with and without FP | Ulcerative stomatitis, obstructive rhinitis-pneumonia complex and FP  14.4% along with Ps. Aeruginosa (non-oxidative pseudomonads) in Green (*Chelonia mydas*) turtle in captivity | The most abundant in individuals with FP in the survey |  | [6] |
| *Pseudomonas aeruginosa* | Central Mediterranean Sea; Florida, USA | * | Green (*Chelonia mydas*), Loggerhead (*Caretta caretta*) | May be from normal flora without pathogenicity;  Opportunistic and able to infect tissues damaged by trauma  ulcerative dermatitis and rhinitis and stomatitis, shell disease, bronchopneumonia in captivity;  Shows 94.1% resistance to antibiotic tested [17] |  | Anthropogenic effects can cause trauma and trigger infection | [5, 11, 17] |
| *Pseudomonas stutzeri* | Florida, USA |  | Green (*Chelonia mydas*) | Pathogenicity not determined. May be from normal flora without pathogenicity |  |  | [5, 6] |
| *Salmonella spp.* | Tortuguero National Park, Costa Rica; Western Australia;  Oman Sea; Persian Gulf | Green (*Chelonia mydas*) |  | Death and illness (enteritis and septicaemia as possible manifestations of reptilian);  May infect eggs;  Resistant to ampicillin | May infect any species (salmonellosis in humans);  Individuals may be carriers | Humans may be the source of infection in captivity | [11, 15, 29] |
| *Salmonella chester* | Australian Northern Territory |  | Green (*Chelonia mydas*) |  | Outbreak of gastroenteritis due to meat consumption of Greens (*Chelonia mydas*);  Salmonella was isolated from partially cooked Green (*Chelonia mydas*) |  | [30] |
| *Salmonella enteritidis /enterica* | Yorke Island/ Eastern Caribbean | Green (*Chelonia mydas*), Hawksbill (*Eretmochelys imbricata)* | Leatherback (*Dermochelys coriacea*) | Diffuse lymphocytic and Foci (<1mm) in liver a of a captive individual  resistant to ampicillin |  |  | [7, 31] |
| *Salmonella* regent |  |  | Hawksbill (*Eretmochelys imbricata*) | Catarrhal colitis |  |  | [29] |
| *Salmonella typhimurium* | West coast of North and Central America; Hawaii | Olive ridley turtle (*Lepidochelys olivacea*) |  | Granulomatous ephritis;  Renal lesions |  |  | [32] |
| *Serratia marcescens* | Canary Islands, Spain |  | Loggerhead (*Caretta caretta*) | Opportunistic pathogen;  May associate with Fibrinous and necrotizing enteritis in a mixed infection |  |  | [3] |
| *Serratia odorifera* | Costa Rica |  | Green (*Chelonia mydas),* Olive ridley (*Lepidochelys olivacea)* | Opportunistic pathogen;  May correlate to hatching failure | May pose risk to egg consumers |  | [18] |
| *Vibrio alginolyticus* | Pacific Ocean; Gulf of California; Canary Islands, Spain | Green (*Chelonia mydas*), Hawksbill (*Eretmochelys imbricata)* | Black (*Chelonia mydas* agassizii), Green (*Chelonia mydas*) with FP, Loggerhead (*Caretta caretta*), Olive ridley (*Lepidochelys olivacea*) | Important cause of mortality;  Ulcerative dermatitis frequently seen; Second frequent bacteria in ulcerative stomatitis lesions;  Exudative bronchopneumonia and/or granulomatous pneumonia, traumatic skin lesions, granulomatous nephritis, renal abscesses, and necrotizing and/or granulomatous hepatitis;  Can infect eggs;  Global spread reported recently particularly clone O3:K6;  No specific treatment, common antibiotics, normally resistant to antibiotics (primarily to ampicillin);  50% of Green (*Chelonia mydas*) with FP in one of the studies | Causing gastroenteritis related to consumption of meat and egg;  Can infect molluscs, crustaceans, fish and cnidarians | Ulcerative dermatitis in boat strike and bycatch;  Humans may be the source of infection in captivity | [3, 25, 33, 34] |
| *Vibrio harveyi* | Hawaii, USA |  | Green (*Chelonia mydas*) | Common in blood samples but no pathogenicity determined | May pose risk to humans;  May infect molluscs, crustaceans and fish |  | [10] |
| *Vibrio tubiashii* | Hawaii, USA |  | Green (*Chelonia mydas*) | Common in blood samples but no pathogenicity determined | May infect molluscs, crustaceans and fish |  | [10] |
| *Vibrio campbelli* | Hawaii, USA |  | Green (*Chelonia mydas*) | Found in blood samples;  No pathogenicity determined | May pose risk to humans;  May infect molluscs, crustaceans and fish |  | [10] |
| *Vibrio parahaemolyticus* | Tuscany, Italy | Black (*Chelonia mydas* *agassizii*), Olive ridley (*Lepidochelys olivacea*) | Loggerhead (*Caretta caretta*) | Fungal and mixed bacterial infection of the skin;  Global warming favors spread;  Treatment: common antibiotics; normally resistant to antibiotics (primarily to ampicillin) | May pose risk to humans | Temperature elevation favors global increase of pandemic V. parahaemolyticus | [20, 25, 35] |
| *Vibrio cholera* |  |  | Black (*Chelonia mydas agassizii*), Olive ridley (*Lepidochelys olivacea*) | Treatment: common antibiotics;  Normally resistant to antibiotics (primarily to ampicillin) susceptible to ciprofloxacin, SXT, tetracycline, and chloramphenicol | *V. cholerae* virulence genes in individuals caught in China | Sea surface temperature can correlate with outbreaks | [35, 36] |
| *Vibrio mimicus* | Costa Rica |  | Olive ridley (*Lepidochelys olivacea)* | Reduced viability of eggs | Egg caused diarrhea in 33 people  Otitis in people who swam in estuarine water |  | [11, 37] |
| *Vibrio damsela* | Eastern coast of Tasmania, Australia |  | Green (*Chelonia mydas*) with and without FP, Leatherback (*Dermochelys coriacea*) | Cause of death for one Leatherback (*Dermochelys coriacea*); endocardia thrombus valvular endocarditis and septicaemia;  GI (gastrointestinal) tract infection led to septicaemia | May infect fish and sharks | Anthropogenic effects caused intestinal lesions | [6, 28] |
| *Vibrio fluvialis* | Hawaii, USA |  | Green (*Chelonia mydas*) with and without FP | Found in samples;  No pathogenicity determined |  |  | [6] |
| *Vibrio aestuarianus* | Hawaii, USA |  | Green (*Chelonia mydas*) with and without FP | Found in blood samples;  No pathogenicity determined |  |  | [10] |

### 4.2. Gram positive bacteria

| Disease hazard | Region reported | Presence in sea turtles | | Outcome of infection (lesion, clinical sign and/or disease)  Symptom in individuals;  Ease of spread, rate of spread;  Diagnostic test or treatment, if available. | Zoonotic/transmissible to companion animals | Correlation with climatic/anthropogenic events | Key reference(s) |
| --- | --- | --- | --- | --- | --- | --- | --- |
|  |  | **Captive populations** | **Wild populations** |  |  |  |  |
| *Bacillus spp.* | Florida, USA and Canary Islands, Spain |  | Green (*Chelonia mydas*) with FP | May be from normal skin flora without pathogenicity;  Fibrinous and necrotizing enteritis;  Ulcerative stomatitis, obstructive rhinitis-pneumonia complex and FP;  May correlate to hatching failure | May pose risk to egg consumers |  | [3, 5] |
| *Corynebacterium spp.* | Thailand | Green (*Chelonia mydas*), Hawksbill (*Eretmochelys imbricata*) |  | May be from normal skin flora without pathogenicity. Opportunistic pathogen |  |  | [13, 38] |
| *Corynebacterium (diphteroids)* | Hawaii, USA |  | Green (*Chelonia mydas*) with and without FP | Non-pathogenic, commensals of skin and upper respiratory tract  Frequent Gram-positive isolation in the survey | May be found in aquatic species |  | [6] |
| *Enterococcus spp.* | East Caribbean Sea; central Mediterranean Sea; Oman sea; Persian Gulf |  | Green (*Chelonia mydas*, Leatherback (*Dermochelys coriacea*), Olive ridley (*Lepidochelys olivacea*) | Opportunistic pathogen. may correlate to hatching failure  Pneumonia, anorexia or poor appetite and lethargy, joint inflammation, radiographic evidence of osteomyelitis and excessive GI tract gas, persistent hypoglycemia, cutaneous wounds and subcutaneous masses  Commercially available test kit, bacteriologic culture of blood, electrophoresis  Once specific *Enterococcus* infection treatment starts, recovery may happen. But no pharmacokinetic studies are available.  May be resistant to ampicillin. | May pose risk to humans | Urban sewages may cause antibiotic resistant if found a way to ocean | [19, 39] |

| *Enterococcus faecalis and faecium* | New England Aquarium Boston, USA; Tuscany, Italy | Leatherback (*Dermochelys coriacea*) | Kemp’s ridley (*Lepidochelys kempii*), Loggerhead (*Caretta caretta*) | Bladder, brain, intestine, kidney, liver, lung and muscle may be infected  Septicemia and osteomyelitis  in sea turtles at the New England Aquarium  Treatment: may be resistant to ampicillin and amoxicillin amoxicillin–clavulanic acid, |  | This bacterium may be introduced through wounds from anthropogenic causes or predation | [20, 39] |
| --- | --- | --- | --- | --- | --- | --- | --- |
| *Lactobacillus spp.* | Hawaii, USA |  | Green (*Chelonia mydas*) with and without FP | A part of microbiota |  |  | [6] |
| *Lactococcus garviae* | Tuscany, Italy |  | Green (*Chelonia mydas*), Loggerhead (*Caretta caretta*) | Detected using PCR;  No pathogenic studies carried out | Present in fish, molluscs and crustaceans;  Identified in a bacterial epidemic in aquatic invertebrates, such as the giant freshwater prawn | Climate change may influence the threat levels associated with such exotic pathogens | [20] |
| *(Lactobacillales) Aerococcus viridans* | Canary Islands, Spain |  | Loggerhead (*Caretta caretta*) | First report of esophageal diverticulum in sea turtles, maybe due to ingestion of infected lobster | Crustaceans |  | [40] |
| *Lysinibacillus fusiformis* | Sugözü Beaches, Turkey | Green (*Chelonia mydas*) |  | Found in egg contents | May be an indicator of pollutants associated with oil in  the nesting area |  | [4] |
| *Micrococcus spp.* |  | * | Green (*Chelonia mydas*) with and without FP | May be from normal skin flora without pathogenicity | Normal flora in marine environments |  | [15] |
| *Staphylococcus spp. (beta-haemolytic)* | Canary Islands, Spain; Costa Rica, Australia; Georgia, USA | Green (*Chelonia mydas*) and Hawksbill (*Eretmochelys imbricata*) | Green (*Chelonia mydas*), Loggerhead (*Caretta caretta*), Olive ridley (*Lepidochelys olivacea*); may contain *S. aureus, S. cromogenes, S. epidermis*, and *S. intermediu* | Normal flora in marine environments but are opportunistic;  Fibrinous exudative pericarditis; ulcerative oesophagitis and stomatitis; gastritis and hepatitis | The most abundant skin-colonising bacteria on human body and the cause of nosocomial infections | Humans may come in contact with eggs and transfer these bacteria or cause resistant to antibiotics | [13, 24, 29, 33, 41] |
| *Staphylococcus aureus* | Eastern Pacific |  | Green (*Chelonia mydas*) without FP, Olive ridley (*Lepidochelys olivacea*) | Common skin flora, but some strains may produce cytotoxins and cause necrotic tissue and pneumonia  May be resistant to Erythromycin |  | Human may come in contact with eggs and transfer these bacteria or cause resistant to antibiotics | [41] |
| *Staphylococcus epidermidis* | Florida, USA | * | Green (*Chelonia mydas*) with and without FP, Olive ridley (*Lepidochelys olivacea*) | Opportunistic and able to infect tissues damaged by trauma  ulcerative dermatitis and rhinitis and stomatitis, shell disease, bronchopneumonia in captivity | Opportunistic and may pose risk to humans | Anthropogenic effects may cause trauma and trigger infection | [5] |
| *Staphylococcus xylosus* | Canary Islands, Spain |  | Loggerhead (*Caretta caretta*) | Pneumonia, Fibrinous exudative pericarditis, nephritis | Cutaneous lesions in chicken, sheep and mice |  | [3] |
| *Staphylococcus lentus* | Tuscany, Italy |  | Loggerhead (*Caretta caretta*) | Detected using PCR. No pathogenic studies carried out | Side-necked (*Phrynops geoffranus*) |  | [20] |
| *Staphylococcus sciuri* | Hawaii, USA |  | Green (*Chelonia mydas*) | Found in blood samples, no pathogenicity determined  Low frequency isolation in the survey |  |  | [10] |
| *Alpha-hemolytic Streptococcus* | Canary Island, Spain; Padre Island, Gulf of Mexico | Green (*Chelonia mydas*) | Green (*Chelonia mydas*) | Opportunistic pathogen  Bilateral Chronic Shoulder Infections (along with Corynebacterium *spp*. and Nocardia *spp*.) in a chelonian mydas Padre Island, TX, USA led to mortality. |  |  | [7, 38] |
| *Non-haemolytic streptococcus* |  |  | Green (*Chelonia mydas*) with and without FP | High frequent isolation in the survey |  |  | [6] |
| *Streptococcus group C* | Thailand | Green (*Chelonia mydas*),  Hawksbill (*Eretmochelys imbricata*) |  | Ulcerative stomatitis along with beta-haemolytic *Staphylococcus* *spp.* |  |  | [13, 15] |

### 4.3. Not defined by gram staining

| Disease hazard | Region reported | Presence in sea turtles | | Outcome of infection (lesion, clinical sign and/or disease)  Symptom in individuals;  Ease of spread, rate of spread;  Diagnostic test or treatment, if available | Zoonotic/transmissible to companion animals | Correlation with climatic/anthropogenic events | Key reference(s) |
| --- | --- | --- | --- | --- | --- | --- | --- |
|  |  | **Captive populations** | **Wild populations** |  |  |  |  |
| *Chlamydia psittaci* | Cayman Turtle Farm, Cayman Islands |  |  | Chlamydiosis systemic infection and evidence of epicarditis, myocarditis, hepatitis splenitis, pneumonia, and nephritis | May infect several species/ known human pathogens | Humans may be the source of infection in captivity | [42, 43] |
| *Mycobacterium avium* | Captured in French Frigate Shoal and rehabilitated at the University of Hawaii, USA | Green (*Chelonia mydas*) |  | Tuberculosis, commonly cutaneous lesions, anorexia, lethargy and wasting;  Focal granulomas characterised by central necrosis seen in liver, lung, | *Mycobacterium* *spp*. may potentially be zoonotic | Humans in captivity or seabirds in nesting beaches may be cause of the infection | [7, 44] |
| *Mycobacterium chelonae* | Adriatic coast of Italy | Kemp’s ridley (*Lepidochelys kempii*) in rehabilitation | Loggerhead (*Caretta caretta*) | Osteolytic lesions, osteoarthritic diseases of synovial joints and systemic disease;  Detection: observation of nodules on internal organs |  | Humans may be the source of infection in captivity | [45, 46] |
| *Mycobacterium haemophilum* | Atlantic coast of Florida | Leatherback (*Dermochelys coriacea*) |  | Disseminated mycobacteriosis mostly affecting the nervous system | May infect various reptiles | Humans may be the source of infection in captivity | [47] |
| *mycobacterial pneumonia* |  | Loggerhead (*Caretta caretta*) |  | Tilted-swimming  respiratory infections are most often fatal and may be contagious;  The bacterial agent wasn’t isolated  resistant to streptomycin  treatment: NMFS STF with 5 mg/kg injectable enrofloxacin |  | Zoonotic | [48, 49] |
| *Dermatophilus chelonae* |  |  | Green (*Chelonia mydas*), Loggerhead (*Caretta caretta*) | Dermatophilosis, Present in a mixed infection leading to cutaneous ulceration along with *V. alginolyticus* |  |  | [11] |
| *Shewanella putrefaciens* | Hawaii, USA |  | Green (*Chelonia mydas*), Loggerhead (*Caretta caretta*) | Low frequency bacteria isolated from Green (*Chelonia mydas*) |  |  | [10] |

### 4.4. Mixed bacterial infections

| Bacteria involved | Region reported | Presence in sea turtles | | Disease | Anthropogenic effect and climatic event | Key reference(s) |
| --- | --- | --- | --- | --- | --- | --- |
|  |  | **Captivity** | **Wild** |  |  |  |
| *Alginolyticus, A. hydrophila, Pseudomonas spp., and Flavobacterium spp.* | Northern Australia | Green (*Chelonia mydas*), Hawksbill (*Eretmochelys imbricata*) | Green (*Chelonia mydas*) | Integumentary, digestive, respiratory;  Traumatic ulcerative dermatitis, ulcerative stomatitis, obstructive rhinitis and bronchopneumonia;  Largely seen in hatchlings and juvenile in captivity | Fishing hooks and boat strike(s) | [7] |
| *Aeromonas hydrophila, Citrobacter*  *spp., Escherichia coli, Proteus spp., Vibrio alginolyticus,*  *and Staphylococcus spp.* |  |  | Loggerhead (*Caretta caretta*) | Granulomatous nephritis and renal abscesses |  | [3] |
| *Klebsiella pneumoniae,*  *Enterobacter agglomerans, E. cloacae, Escherichia coli, Klebsiella oxytoca, K pneumoniae, and Serrana (serratia) marcescens* | Nesting beach of Costa Rica |  | Green (*Chelonia mydas*) | Egg failure |  | [24] |
| *Pseudomonas aeruginosa and Flavobacterium sp* | Northern Australia |  | Green (*Chelonia mydas*) | Eyelids: ulcerative lesions |  | [7] |
| *Bacillus sp Escherichia coli, Pasteurella spp., Proteus spp., Serratia marcescens, Staphylococcus spp., Streptococcus spp., and Vibrio alginolyticus* | Canary Islands, Spain; East Caribbean Sea |  | Green (*Chelonia mydas*) with and without FP,  Leatherback (*Dermochelys coriacea*), Loggerhead (*Caretta caretta*) | Digestive lesions exudative bronchopneumonia and/or granulomatous pneumonia | Fishing hooks and boat strike(s) | [34] |
| *Aeromonas hydrophila, Citrobacter spp., Escherichia coli, Proteus spp., Staphylococcus spp., and Vibrio alginolyticus infections* | Canary Islands, Spain |  | Green (*Chelonia mydas*), Leatherback (*Dermochelys coriacea*), Loggerhead (*Caretta caretta*) | Necrotizing and/or granulomatous hepatitis |  | [27] |
| *Burkholderia cepacia, Pseudomonas spp., Staphylococcus spp., and Achromobacter spp.* | Canary Islands, Spain |  | Loggerhead (*Caretta caretta*) | Heterophilic scleritis |  | [3] |
| *Serratia mareescens* (and *Aeromonas spp., Bacillus spp., Enterobacter spp., Escherichia coli, Klebsiella spp., Pasteurella spp., Proteus spp., Pseudomonas spp., Serratia marcescens, Staphylococcus spp., and Vibrio spp.)* | Rio de Janeiro, Brazil | * | Green (*Chelonia mydas*) | Bronchopneumonia, integumental lesions, obstructive rhinitis, traumatic ulcerative dermatitis, ulcerative shell disease, and ulcerative stomatitis;  Abscesses of the salt-secreting gland and peritoneal wall | Fishing hooks and boat strike(s) | [24] |

## S5. Fungi

| Disease hazard | Region reported | Presence in sea turtles | | Outcome of infection (lesion, clinical sign and/or disease)  Symptom in individuals;  Ease of spread, rate of spread;  Diagnostic test or treatment, if available | Zoonotic/transmissible to companion animals | Correlation with climatic/anthropogenic events | Key reference(s) |
| --- | --- | --- | --- | --- | --- | --- | --- |
|  |  | **Captive populations** | **Wild populations** |  |  |  |  |
| *Acremonium spp.* | Shoalwater, Heron Reef, Peak Island, Australia |  | Flatback (*Natator depressus*), Green (*Chelonia mydas*), Loggerhead *(Caretta caretta)* | Isolated from nesting females’ cloaca | May rarely infect humans | Isolated in ocean sediments and at 30°C;  Such isolations were halotolerant and even halophilic | [50, 51] |
| *Allescheria spp.* | Nancite, Costa Rica |  | Olive ridley (*Lepidochelys olivacea*) | Isolated from failed eggs shell and chamber | May infect immunosuppressed hosts |  | [52] |
| *Alternata arborescens* | Rio  Grande do Sul, Southern Brazil |  | Loggerhead *(Caretta caretta)* | Phaeohyphomycosis, kidney nodules, peritonitis and nephritis (along with *C. cladosporioides*) | Emerging opportunistic  mycotic infections agents of domestic animals |  | [53] |
| *(Pseud)allescheria boydii* | Heron lsland, Wreck Island, Peak Island, Mon Repos, Milman Island, Australia |  | Flatback (*Natator depressus*), Green (*Chelonia mydas*), Hawksbill (*Eretmochelys imbricata*), Loggerhead *(Caretta caretta)* | Found in failed eggs |  |  | [54] |
| *Aspergillus spp.* | Turkey; Pacific coast of Costa Rica |  | Green (*Chelonia mydas*),  Kemp’s ridley (*Lepidochelys kempii*), Loggerhead *(Caretta caretta),* Olive ridley (*Lepidochelys olivacea*) | Skin diseases in captive individuals;  Airborne infection and may be present in successful nests. Has been found in many failed nests of different individuals;  Focal, dry, black areas on flippers of hatchlings;  Necrotic lesions on head, neck and shell of Kemp’s ridley (*Lepidochelys olivacea*);  Potential treatment Fluconazole topical iodine | Immunosuppressed individuals | May produce  Immunosuppressive mycotoxins, such as gliotoxin at higher temperatures;  Water pollution may trigger infection | [22, 41, 55, 56] |
| *Aspergillus niger* | Woongarra Coast, Australia |  | Loggerhead *(Caretta caretta)* | Isolated from egg chamber |  |  | [41] |
| *Absidia* | Fethiye, Turkey; Nancite, Costa Rica |  | Loggerhead *(Caretta caretta)*, Olive ridley (*Lepidochelys olivacea*) | Isolated from nest – may correlate with hatching failure | An emerging cause of mucormycosis | *Absidia* may correlate with nitrification increase (greenhouse gas production) | [56-58] |
| *Candida albicans* | Canary Islands, Australia |  | Loggerhead *(Caretta caretta)* | Systemic mycotic infection and Intestinal candidiasis;  Potential treatment Fluconazole | Immunosuppressed human and animal |  | [3, 34] |
| *Cephalosporium sp* | USA | Kemp’s ridley (*Lepidochelys*  *kempii*) |  | Necrotic lesions on the head, neck, and shell | Plant pathogen |  | [48] |
| *Cephalosporium*  *curtipes var.*  *uredinicola* | Woongarra Coast, Australia |  |  | Isolated from failed eggs |  |  | [41] |
| *Cladosporium sp* | Cayman Turtle Farm, Cayman Islands; Yanıklar and Fethiye, Turkey; Woongarra Coast, AUS; Heron Reef, North West Island, Australia; Playa Grande, Costa Rica | Green (*Chelonia mydas*) | Green (*Chelonia mydas*), Loggerhead (*Caretta caretta),*  Olive ridley (*Lepidochelys olivacea*) | Has been isolated from egg chamber, egg shell and cloacal fluid. May correlate with hatching failure;  Pneumonic lesions in captivity | Immunosuppressive sea individuals | Prefer lower  Temperatures;  The infection is more probable in popular beaches (anthropogenic effects) | [41, 50, 59] |
| *Cladosporium cladosporioides* | Rio  Grande do Sul, Southern Brazil |  | Loggerhead *(Caretta caretta)* | Phaeohyphomycosis, Kidney Nodules, peritonitis and nephritis (along with *Alternata arborescens*) | Emerging opportunistic  mycotic infections agents of domestic animals |  | [53] |
| *Colletotrichum acutatum* | Florida, USA | Kemp’s ridley (*Lepidochelys*  *kempii*) | Kemp’s ridley (*Lepidochelys*  *kempii*) | Mycotic nephritis, pneumonia, granulomatous hepatitis and granulocytic hyperplasia of the bone marrow of the carapace;  Immunocompromised juvenile Kemp’s ridley (*Lepidochelys*  *kempii*) | Very rarely known to cause disease in humans |  | [60] |
| *Chrysosporium* | Yanıklar and Fethiye, Turkey |  | Loggerhead *(Caretta caretta)* | Frequently isolated from sand and egg shell of failed eggs | Emerging infection in snakes [61] | Isolated from nails.  Handling the eggs may cause infection | [56, 61] |
| *Cunninghamella* | Nancite, Costa Rica; East Australia |  | Olive ridley (*Lepidochelys olivacea*) | Isolated from failed eggs shell and chamber |  |  | [50, 52] |
| *Cylindrocarpon* | Yanıklar and Fethiye, Turkey |  | Loggerhead *(Caretta caretta)* | Isolated from nest chamber of failed eggs |  |  | [56] |
| *Drechslera spp.* |  |  | Hawksbill (*Eretmochelys imbricata*) | Necrotic lesions on the head, neck, and shell | Isolated from sea birds’ cloaca but haven’t been isolated from eggs |  | [54, 62] |
| *Emericella* | Yanıklar and Fethiye, Turkey |  | Loggerhead *(Caretta caretta)* | Isolated from nest chamber of failed eggs |  |  | [56] |
| *Eretmochelys imbricata* |  |  | Kemp’s ridley (*Lepidochelys kempii)* | Lung, liver and kidney infection along with *Colletotrichum acutatum* |  |  | [60] |
| *Geotrichum spp.* | Philippines; Playa Grande, Costa Rica | Hawksbill (*Eretmochelys imbricata*) | Loggerhead *(Caretta caretta)*, Olive ridley (*Lepidochelys olivacea*) | Necrotic lesions on the head, neck, and shell;  Isolated from cloacal fluid and may correlate with hatching failure | Immunocompromised | Cooler temperatures, between 25-37°C increase the growth rate | [41, 62] |
| *Gliocladiopsis* | Nancite, Costa Rica |  | Olive ridley (*Lepidochelys olivacea*) | Isolated from failed eggs |  |  | [52] |
| *Fusarium spp.* | Playa Grande, Costa Rica; St Croix, U.S. Virgin  Islands; Raine Island, Australia; Isla de la Plata at Machalilla National Park, Ecuador | Hawksbill (*Eretmochelys imbricata*), Kemp’s ridley (*Lepidochelys kempii)* | Leatherback (*Dermochelys coriacea*), Loggerhead (*Caretta caretta*), Olive ridley (*Lepidochelys olivacea*) | Dermatomycosis (normally shell is infected) necrotic skin lesions more often in captivity;  Pneumonic lesion  Known fungus of nest chamber; Probably infects a non-viable egg before spreading to other viable eggs  Is suggested to be considered for differential diagnosis of shell and skin lesions in sea turtles | Failed eggs of all species | Non-halophiles at 25ºC and 30°C | [29, 41, 51, 63-65] |
| *Fusarium oxysporum* | Columbia; Heron lsland, Wreck Island, Peak Island, Mon Repos, Milman Island, Australia |  | Flatback (*Natator depressus*),  Hawksbill (*Eretmochelys imbricata*), Leatherback (*Dermochelys coriacea*) | Excrete mycotoxins in eggs |  |  | [55] |
| *Fusarium scirpi* | Northern Australia | Green (*Chelonia mydas*) |  | Pneumonic lesions in captivity |  |  | [7] |
| *Fusarium solani* | Bahamas; Spain;  Columbia; Woongarra Coast, Heron lsland, Wreck Island, Peak Island, Mon Repos, Milman Island, Australia; Boavista, Cape Verde | Loggerhead (*Caretta caretta)* | Flatback (*Natator depressus*), Green (*Chelonia mydas*), Hawksbill (*Eretmochelys imbricata*), Kemp’s ridley (*Lepidochelys kempi)*,  Leatherback (*Dermochelys coriacea*), Loggerhead (*Caretta caretta)* | Skin diseases in captive individuals;  Cutaneous abscess in Kemp’s ridley (*Lepidochelys kempii)*  mass mortalities in natural and relocated nests (excrete  mycotoxins in eggs);  100% mortality of Loggerhead (*Caretta caretta)* nests in Boavista, Cape Verde;  May have an effect on hatchlings fitness | Immunosuppressed individuals;  Potentially zoonotic |  | [63, 64, 66, 67] |
| *Fusarium falciforme, Fusarium keratoplasticum* | Raine Island, Australia |  | Green (*Chelonia mydas*) | Normally seen is nesting beaches, reduce hatching success (able to kill up to 90% of the embryos) | Able to infect stressed individuals |  | [64] |
| *Homodendrum* | Nancite, Costa Rica |  | Olive ridley (*Lepidochelys olivacea*) | Isolated from failed egg shells and chamber |  |  | [52] |
| *Mucor spp.* | Göksu Delta, Turkey; Yanıklar and Fethiye, Turkey;  Nancite, Costa Rica; Heron Island, Australia |  | Green (*Chelonia mydas)*, Loggerhead *(Caretta caretta)*, Olive ridley (*Lepidochelys olivacea*) | May correlate with hatching failure | An emerging cause of *mucormycosis* | Temperatures lower than 37°C favours its growth | [41, 56, 58] |
| *Paecilomyces spp.* | Australia | Kemp's ridley (*Lepidochelys*  *kempii*), Green (*Chelonia mydas)* | Green (*Chelonia mydas*), Olive ridley (*Lepidochelys olivacea*) | Granulomatous pneumonia  necrotic lesions on the head, neck, and shell;  Detection: histopathology and culture | May infect humans |  | [48] |
| *Paecilomyces*  *lilacinus (Purpureocillium lilacinum)* | Woongarra Coast, Australia; Cayman Turtle Farm, Cayman Islands | Green (*Chelonia mydas*),  Loggerhead (*Caretta caretta)*  Is found in loggerhead hatchlings too | Hawksbill (*Eretmochelys imbricata*), Loggerhead *(Caretta caretta)* | Purpureocilliosis  disseminated granulomas  buoyancy abnormality and pneumonia in captivity leading to death (Detected using histopathology after necropsy);  hatchling skin and pulmonary lesions  Isolated from failed eggs | Immunosuppressed humans or animals. Has been reported in several cases of reptiles and also chelonians |  | [59, 68-70] |
| *Penicillium spp.* | Yanıklar and Fethiye, Turkey; Mon Repos, Australia; Peak Island, Milman Island, Australia; Nancite, Costa Rica |  | Flatback (*Natator depressus*), Hawksbill (*Eretmochelys imbricata*), Loggerhead (*Caretta caretta*), Olive ridley (*Lepidochelys olivacea*) | Skin diseases in captive individuals;  Detection: culture;  (pneumonic lesions) lung is affected, dissemination of other organs is possible;  May correlate to hatching failure | Turtles and tortoises | Grow best at temperatures below 37°C; halotolerant at 25°C | [29, 41, 50, 51] |
| *Phialophora spp.* | Peak Island, Australia |  | Flatback (*Natator depressus*) | Isolated from cloacal samples of interesting turtles |  |  | [50] |
| *Rhodotorula spp.* | Northern Australia |  | Green (*Chelonia mydas*) | Bronchopneumonia in captivity | Various sea turtle species;  May infect humans |  | [7] |
| *Saksenaea vasiformis* | Nancite, Costa Rica |  | Olive ridley (*Lepidochelys olivacea*) | Isolated from failed egg shells and chamber |  |  | [52] |
| *Scedosporium*  *aurantiacum* | Woongarra Coast, Australia |  | Loggerhead *(Caretta caretta)* | Isolated from failed eggs |  |  | [41] |
| *Scolecobasidium constrictum* | USA | Kemp’s ridley (*Lepidochelys*  *Kempii)* | Hawksbill (*Eretmochelys imbricata*); | Skin diseases in captive sea turtles;  Pulmonary mycoses infections  necrotic lesions on the head, neck, and shell |  |  | [48] |
| *Sporotrichium*  *spp.* | Cayman Turtle Farm, Cayman Islands | Green (*Chelonia mydas*) |  | Pneumonic lesions and buoyancy abnormality along with *Cladosporium spp*., and *Paecilomyces spp.* |  |  | [59] |
| *Thielavia* | Yanıklar and Fethiye, Turkey |  | Loggerhead *(Caretta caretta)* | Isolated from egg shells, may correlate with hatching failure |  |  | [56] |
| *Trichophyton spp.* | Canary Islands, Spain |  | Olive ridley (*Lepidochelys olivacea*) | Ulcerative lesions on flipper, pneumonia | Cause forms of dermatophytosis in humans |  | [33] |
| Unknown Fungi |  | Hawksbill (*Eretmochelys imbricata*) |  | Crusty yellow lesions on neck and flippers;  Congestion of intestinal blood vessels |  |  | [71] |

## S6. Parasites

| Disease hazard | | Region reported | Presence in sea turtles | | Outcome of infection (lesion, clinical sign and/or disease)  Symptom in individuals;  Ease of spread, rate of spread;  Diagnostic test or treatment, if available | Zoonotic/transmissible to companion animals | Correlation with climatic/anthropogenic events | Key reference(s) |
| --- | --- | --- | --- | --- | --- | --- | --- | --- |
|  |  |  | **Captive populations** | **Wild populations** |  |  |  |  |
| Protozoa | | | | | | | | |
| *Caryospora cheloniae* and a genotype most closely related to species of *Schellackia* | | Grand Cayman Island, Cayman Islands; Queensland, Australia; Canary Islands, Spain; north-western Africa | Green (*Chelonia mydas)* | Green (*Chelonia mydas),*  Leatherback *(Dermochelys coriacea)*, Loggerhead (*Caretta caretta*) | Coccidiosis  Granulomatous encephalitis, enteritis and, thyroiditis and nephritis; gastritis mass mortalities in South-East Queensland, Australia;  Detection: histopathology, faecal flotation, blood smear and buffy coat examination | Not zoonotic |  | [3, 72, 73] |
| *Cryptosporidium parvum* | | Oahu and the western shores of Maui, Hawaii, USA |  | Green (*Chelonia mydas*) | No correlation was found to stranding;  Detection from faecal and intestinal samples (culture and microscopically) | Possibly zoonotic;  Turtles may carry the oocytes and infect humans; emerging food borne pathogen | Raw sewage disposal may cause the pollution | [26, 74] |
| *Eimeria carettae* | | Martin County, Florida, USA; Atlantic Sea |  | Loggerhead *(Caretta caretta)* | Oocysts were found in the faeces, but no correlation was found with stranding |  |  | [75] |
| *Entamoeba*  *invadens* | |  | Green (*Chelonia mydas)*, Loggerhead *(Caretta caretta)* | Leatherback *(Dermochelys coriacea)* | Mortality in hatchlings in captivity | Possibly zoonotic;  Turtles may be sub-clinical carriers of amoebiasis |  | [26] |
| Metazoa | | | | | | | | |
| Nematodes | | | | | | | | |
| Family/Genus | **Species** |  | | | | | | |
| *Anisakis spp.* | -- | Canary Islands, Spain; Western Australia, Australia | * | Loggerhead (*Caretta caretta*) | Ulcer in stomach and intestine, dominant between helminths;  Granulomatous hepatic serositis;  Hemorrhagic and ulcerative disease resulting from larval migration;  Samples are collected during necropsy and gross examination | Zoonotic  A wide range of marine hosts;  Sea turtles may act as accidental or paratenic hosts | Captive turtles were fed by fresh sardines (the reason of infection) | [3, 76] |
|  | *Anisakis pegreffii* | Central Mediterranean of southern  Italy |  | Loggerhead (*Caretta caretta*) | Samples are collected during necropsy and gross examination;  Detection: PCR and histochemistry. |  |  | [76] |
|  | *Sulcascaris sulcata* | Brazil; Mediterranean Sea; Western Pacific; Australia; Uruguay; Atlantic Ocean; Florida, USA |  | Green (*Chelonia mydas)*, Loggerhead (*Caretta caretta*) | Esophagus and small intestine may be infected | Loggerhead (*Caretta caretta*), as a carnivore, is the main host;  Mediterranean mussel (*Mytilus galloprovincialis*) is the intermediate host;  Can infect edible scallops |  | [76-79] |
| *Cucullanidae* | *Cucullanus cauettae* | Western Australia; Mediteranean Sea |  | Loggerhead (*Caretta caretta*) | Were found in intestine |  |  | [76] |
| *Echinocephalus spp.spp.* |  | Western Mediterranean |  | Loggerhead (*Caretta caretta*) | Immature worms | Main host: elasmobranchs |  | [80] |
|  | *Hysterothylacium* | Adriatic Sea |  | Loggerhead (*Caretta caretta*) | Generalist helminth found with a low frequency |  |  | [81] |
| *Gnathostomatidae* | *larval gnasthostome Echinocephalus sp* | Shark Bay, Australia |  | Green (*Chelonia mydas),* Loggerhead (*Caretta caretta*) |  |  |  | [82] |
| *Kathlanidae* | *Kathlania leptura* | Brazil; Egypt; Georgia, USA; Sri Lanka; Mauritania; Mediterranean island; Ossabaw Island, USA; Western Australia, Australia |  | Green (*Chelonia mydas*), Loggerhead (*Caretta caretta*), Olive ridley (*Lepidochelys olivacea*) | Large intestine may be infected. Infections may be intense | Bivalves, cephalopods, crustaceans, and fish |  | [77, 82] |
|  | *Tonaudia tonaudia* | Costa Rica |  | Green (*Chelonia mydas),* Loggerhead (*Caretta caretta*), Olive ridely (*Lepidochelys olivacea*) | Were found gastrointestinal tract |  |  | [83] |
| *Oxyuridae spp.spp.* |  | Western Mediterranean |  | Loggerhead (*Caretta caretta*) | Immature worms |  |  | [80] |
| Taxon: Platyhelminthes | | | | | | | | |
| *Pronopsis psenopsis* | | Western Mediterranean |  | Olive ridely (*Lepidochelys olivacea*) | Adult worms |  |  | [80] |
| Sub taxon: Trematodes | | | | | | | | |
| *Angiodictyidae* | *Deuterobaris intestinalis* | Costa Rica |  | Green (*Chelonia mydas*) | Were found in intestine; specialist parasites |  |  | [84] |
|  | *Microscaphidium reticulare* | Costa Rica |  | Green (*Chelonia mydas)* | The second most prevalent and the most abundant trematode in the study; only immature parasites were found in; were found in intestine; specialist parasites | Were found in birds and fish | Stress may trigger the infection | [84] |
|  | *Microscaphidium warui* | Costa Rica |  | Green (*Chelonia mydas*) | only immature parasites were found in; were found in urinary bladder; specialist parasites | Were found in birds and fish | Stress may trigger the infection | [84] |
|  | *Octagium hyphalum (hiphalum)* | Queensland, Australia; Costa Rica |  | Green (*Chelonia mydas)* | Only immature parasites were found in Costa Rica; Were found in large intestine | Were found in birds and fish | Stress may trigger the infection | [84] |
|  | *Polyangium linguatula* | Costa Rica |  | Green (*Chelonia mydas*) | Were found in intestine |  |  | [84] |
| *Aspidogastridae* | *Lophotaspis*  *vallei* | Costa Rica |  | Loggerhead (*Caretta caretta*) | The only non-digenean trematode reported. Were found oesophagus and stomach |  |  | [83] |
| *Brachycoeliidae* | *Cymatocarpus solearis* | Brazil; Adriatic Sea and Northern Ionian Sea |  | Hawksbill (*Eretmochelys imbricata*), Loggerhead (*Caretta caretta*) | Were found in stomach and detected in faeces |  |  | [85, 86] |
| *Calycodidae Family* | *Calycodes anthos* | Brazil; Adriatic Sea; East, Central and Western Mediterranean; Portugal |  | Loggerhead (*Caretta caretta*) | Were found in large intestine; Host specific; low frequency and only in small juveniles |  |  | [77, 81, 87] |
| *Clinosomatidae* | *Clinostomum complanatum* | Caribbean Sea, Costa Rica |  | Green (*Chelonia mydas*) | Only immature parasite found in oesophagus; specialist parasites | A generalist in birds |  | [84] |
| *Hemiuroidea sp* |  | West Mediterranean |  | Loggerhead (*Caretta caretta*) | Found in stomach and intestine, but GI tract is probably having a defence against them (only sexually immature parasite found) | Fish parasites probably recruited through shared prey |  | [88] |
| *Pachypsolidae* | *Pachypsolus irroratus* | Adriatic Sea; East, Central and Western Mediterranean |  | Loggerhead (*Caretta caretta*) | Were found in stomach and intestine; were frequently found in heavy infections and throughout all classes of turtles.  Infects juveniles the most;  Host specific |  |  | [81, 88] |
| *Paramphistomidae* | *Schizamphistomoides erratum* | Costa Rica |  | Green (*Chelonia mydas*) | Were found in large intestine |  |  | [84] |
|  | *Schizamphistomoides scleroporum* | Costa Rica |  | Green (*Chelonia mydas)* | Were found in intestine and stomach |  |  | [84] |
| *Plagiorchiidae* | *Enodiotrema megachondrus* | Central and Western Mediterranean; North-eastern Atlantic |  | Loggerhead (*Caretta caretta*) | Host specific; The dominant species in Western Mediterranean and North-eastern Atlantic;  Were detected in faeces |  |  | [81, 86, 88] |
| *Pronocephalidae* | *Adenogaster serialis* | East Mediterranean |  | Green (*Chelonia mydas*) | Were found in intestine |  |  | [88] |
|  | *Charaxicephaloides spp.spp.* | Costa Rica |  | Green (*Chelonia mydas)* | Were found in stomach |  |  | [84] |
|  | *Charaxicephalus robustus* | Costa Rica |  | Green (*Chelonia mydas*) | Were found in intestine and stomach |  |  | [84] |
|  | *Cricocephalus albus* | Coast of Espírito Santo, Brazil |  | Green (*Chelonia mydas),* Hawksbill (*Eretmochelys imbricata*) | Were found in oesophagus, stomach, small and large intestine | Main host: sea turtle |  | [85, 89] |
|  | *Cricocephalus resectus* | Costa Rica |  | Green (*Chelonia mydas*) | Were found in oesophagus and stomach | Occasionally been isolated from the marine French angelfish |  | [84] |
|  | *Cricocephalus megastomus* | Costa Rica |  | Green (*Chelonia mydas)* | Were found in oesophagus and stomach |  |  | [84] |
|  | *Desmogonius desmogonius* | Costa Rica |  | Green (*Chelonia mydas*) | Were found in oesophagus and stomach; specialist parasites |  |  | [84] |
|  | *Diaschistorchis pandus* | East Mediterranean; Brazil |  | Hawksbill (*Eretmochelys imbricata*), Loggerhead (*Caretta caretta*) | Were found in small intestines |  |  | [85, 88] |
|  | *Himasomum lobatus* | Costa Rica |  | Green (*Chelonia mydas*) | Were found in intestine; generalist |  |  | [84] |
|  | *Metacetabulum invaginatum* | Brazil |  | Hawksbill (*Eretmochelys imbricata*) | Were found in small intestine |  |  | [85] |
|  | *Pleurogonius longiusculus; P. linearis; P. sindhii; P. solidus* | Costa Rica |  | Green (*Chelonia mydas*) | Were found in intestine |  |  | [84] |
|  | *Pleurogonius trigonocephalus* | East, Central and Western Mediterranean; Egypt |  | Loggerhead (*Caretta caretta*) | Were found in intestine |  |  | [81, 88] |
|  | *Pronocephalus obliquus* | Brazil |  | Hawksbill (*Eretmochelys imbricata*) | Were found in stomach and high intestine; specific to the host |  |  | [85] |
|  | *Pyelosomum cochlear* | Costa Rica |  | Green (*Chelonia mydas)* | Infected more than 50% of the studied sea turtles; were found in urinary bladder |  |  | [84] |
|  | *Pyelosomum renicapite* | Brazil, Portugal |  | Loggerhead (*Caretta caretta*) | Were found in large intestine |  |  | [77, 87, 88] |
|  | *Rameshwarotrema uterocrescens* | Costa Rica |  | Green (*Chelonia mydas)* | Were found in oesophagus; generalist |  |  | [84] |
| *Rhytidodidae* | *Rhytidodes gelatinosus* | Adriatic Sea; Central and Western Mediterranean; Egypt |  | Loggerhead (*Caretta caretta*) | Were found in stomach and intestine; mostly infecting juveniles |  |  | [81, 88] |
|  | *Rhytidodoides intestinalis* | Costa Rica |  |  | Were found in gall bladder; specialist parasites |  |  | [84] |
|  | *Rhytidodoides similis* | Costa Rica |  |  | Were found in gall bladder and liver; specialist parasites |  |  | [84] |
| *Spirochidae* family | | USA; Australia; India; Pakistan |  | Various sea turtle species in Australia in last 25 years with a prevalence between 41% and 98% | Important cause of stranding and mortality in sea turtles worldwide (up to 40% mortality in Australia);  Obstruction of blood vessels; normally cardiovascular and gastrointestinal system are affected. In heavy infestations bronchopneumonia and septicemia–toxemia is also reported;  Tissue damages are open doors for secondary bacterial infections (*Salmonella, Escherichia coli, Citrobacter, and Moraxella spp.*);  Detection: Histopathological studies of affected tissues | Not zoonotic |  | [49, 90, 91] |
| *Spirocozohiae* family | *Amphiorchis caborojoensis* | Brazil |  | Hawksbill (*Eretmochelys imbricata*) | Found in body wash |  |  | [85] |
|  | *Amphiorchis solus Simha and Chattopadhyaya* | Costa Rica |  | Green (*Chelonia mydas)* | Found in intestine |  |  | [84] |
|  | *Carettacola, Hapalotrema* | Atlantic seaboard (Florida to Massachusetts), USA |  | Green (*Chelonia mydas*), Loggerhead (*Caretta caretta*) | Acute inflammatory response in intestine (blood fluke eggs) along with Neospirorchis |  |  | [90] |
|  | *Carettacola hawaiiensis* | Hawaii, USA; Australia |  | Green (*Chelonia mydas*), Loggerhead (*Caretta caretta*) | Blood flukes in hepatic vessels. Gross lesions included variously sized, lobulated tumours, serous atrophy of fat, and oedema in the subcutaneous tissues and in the pectoral and coracoid muscles |  |  | [92] |
|  | *Carettacola stunkardi* | Brazil; USA; Panama |  | Green (*Chelonia mydas*), Hawksbill (*Eretmochelys imbricata*) | Found in liver and body wash |  |  | [85] |
|  | *Haplotrema spp.* | Australia; Taiwan; Florida, USA |  | Green (*Chelonia mydas*), Hawksbill (*Eretmochelys imbricata*),  Loggerhead (*Caretta caretta*) | Blood flukes from heart and major vessels;  Granulomata along with *Learedius spp.spp*.  Severe enteric lesions in Loggerhead (*Caretta caretta*) |  |  | [93] |
|  | *Hapalotrema dorsopora* | Australia; Hawaii, Atlantic seaboard (Florida to Massachusetts), USA |  | Green (*Chelonia mydas*); Loggerhead (*Caretta caretta*) | Blood flukes from heart and major vessels;  Eggs were recovered from intestine scraping |  |  | [90, 92] |
|  | *Hapalotrema mistroides* | Adriatic Sea and Northern Ionian Sea |  | Loggerhead (*Caretta caretta*) | Were detected in faeces |  |  | [86] |
|  | *Hapalotrema postorchis* | Costa Rica |  | Green (*Chelonia mydas)* | May infect great vessels and heart;  Specialist parasite |  |  | [84] |
|  | *Haemoxenicon spp.* | Atlantic seaboard, USA |  | Loggerhead (*Caretta caretta*) | The eggs were found in sea turtles |  |  | [90] |
|  | *Learedius learedi* | Bermudan waters,  Baja California, Mexico; Australia; Hawaii, Atlantic seaboard (Florida to Massachusetts) |  | Black, (*Chelonia mydas agassizii*), Green (*Chelonia mydas*), Loggerhead (*Caretta caretta*) | Adult nematodes;  Blood flukes in heart chamber and major blood vessels (Cardiovascular spirorchidiasis along with infection with *Hapalotrema dorsopora* and *Carettacola hawaiiensis*)  Were found in great vessel; heart; oesophagus; gall bladder; liver and intestine;  Eggs were recovered from intestine scraping;  Generalist parasite;  The most prevalent was 97.5% in Costa Rica |  |  | [84, 90, 92] |
|  | *Metacetabulum invaginatum* | Coast of Espírito Santo, Brazil |  | Green (*Chelonia mydas*) |  |  |  | [89] |
|  | *Monticellius indicum* | Costa Rica;  coast of Rio de Janeiro, Brazil |  | Green (*Chelonia mydas)*, Loggerhead (*Caretta caretta*) | Were found in heart; specialist parasite;  GI tract, endocrine system and heart of a Loggerhead (*Caretta caretta)* |  |  | [84, 94] |
|  | *Neoctangium travassosi* | Queensland, Australia; Puerto Rico; coast of Espírito Santo, Brazil |  | Green (*Chelonia mydas*), Hawksbill (*Eretmochelys imbricata*) | Found in GI tract |  |  | [89, 95] |
|  | *Octangium sagitta* | Queensland, Australia |  | Green (*Chelonia mydas)* | Found in GI tract |  |  | [95] |
|  | *Neospirorchis spp.spp. And schistosomatoides* | Costa Rica |  | Green (*Chelonia mydas*) | Adult nematodes |  |  | [83] |
|  | *Monticellius* | Atlantic seaboard (Florida to Massachusetts), USA |  | Loggerhead (*Caretta caretta*) | Eggs were recovered from intestine scraping |  |  | [90] |
| *Styphlotrematidae* | *Styphlotrema solitaria* | West Mediterranean; Brazil; Florida, USA; Costa Rica |  | Hawksbill (*Eretmochelys imbricata*), Loggerhead (*Caretta caretta*) | Were found in stomach, small and large intestine | A generalist species found only in sea turtles |  | [85, 88, 96] |
| *Telorchiidae* | *Orchidasma amphiorchis* | Brazil; Oaxaca, Mexico; Italy; Florida, USA; Australia; Adriatic Sea; Japan; Mediterranean Sea |  | Green (*Chelonia mydas*), Hawksbill (*Eretmochelys imbricata*), Loggerhead (*Caretta caretta*) | Most frequent species, 897 parasites in 18 individuals;  Found in small intestine. Infections may be intense | Main host: sea turtles and teleosts |  | [81, 85, 88] |
| Cestodes | | | | | | | | |
| *Ancistrocephalus imbricatus* | | Western Mediterranean |  | Loggerhead (*Caretta caretta*) | Found in stomach and intestine | Main host: teleosts |  | [80] |
| *Nybelinia spp.spp.* | | Western Mediterranean |  | Loggerhead (*Caretta caretta*) |  |  |  | [88] |
| *Tentacularia coryphaenae* | | Western Mediterranean |  | Green (*Chelonia mydas*), Loggerhead (*Caretta caretta*) | Found in stomach and intestine; Larvae | Main host: elasmobranchs |  | [80] |
| *Trypanorhynchidae* | *Lacistorhynchus/Eutetrarhynchus* | Egyptian Coast |  | Loggerhead (*Caretta caretta*) | Larvae | Main host: elasmobranchs |  | [83] |
| Acanthocephalan | | | | | | | | |
| *Bolbosoma spp.spp.* | | Western Mediterranean; Portugal |  | Loggerhead (*Caretta caretta*) |  |  |  | [87, 88] |
| *Rhadinorhynchus pristis* | | Western Mediterranean; Portugal |  | Loggerhead (*Caretta caretta*) | Were found in intestine (only found in 1 location) |  |  | [87, 88] |
| Anelides | | | | | | | | |
| *Diplotesticulata, Oligochaeta* | | Turkey |  |  | Found in sand and empty egg shells |  |  | [97] |
| *Hydroides spp.spp. and Loimia spp.spp.* | | Brazil |  | Hawksbill (*Eretmochelys imbricata*) |  |  |  | [29] |
| *Ozobranchus branchiatus* | | Caribbean coast; Atlantic Ocean; Costa Rica; Australia; Pacific coast of Mexico; Hawaii, Florida, North Carolina, USA |  | Green (*Chelonia mydas*), Loggerhead (*Caretta caretta*), Olive ridley (*Lepidochelys olivacea*) | Found on the skin around the throat, dorsal surface of the neck, and axillary  region of the flippers;  may cause severe skin lesions,  deep cutaneous erosion, eye injuries and even host death;  Generalist parasite;  Possible vector for FP (Fibropapillomatosis) herpes viruses | May complete the reproductive cycle on sea turtles |  | [22, 83, 98] |
| *Ozobranchus margoi* | | Hawaii, USA; Rio Grande do Sul, Brazil; Tobago, West Indies |  | Green (*Chelonia mydas*), Loggerhead (*Caretta caretta*) | Possible vector for FP herpes viruses;  May cause severe skin lesions, deep cutaneous erosion, eye injuries and even host death;  Detection: visual inspection of external surfaces | Not zoonotic;  May complete the reproductive cycle on sea turtles |  | [22, 98] |
| Arthropodes | | | | | | | | |
| *Arachnida (Archnoidea) mites (Acaridae)* | *Rizoglyphus spp.* | Mexico |  | Leatherback (*Dermochelys coriacea*) | Mites embedded in skin  The correlation with hatching failure is not clear  Detection: uncertain | Unknown if it is able to cause diseases in humans |  | [99, 100] |
| *Chelonibiidae and Platylepadidae* | *Chelonibia spp., Platylepas spp.* | Japan; Mexico; Atlantic Ocean; Mediterranean; Florida, USA |  | Loggerhead (*Caretta caretta*) | External barnacles attached to carapace and plastron, species are different over larger expanse of space. | Not able to cause diseases in humans |  | [83] |
| *Coleoptera* | *Cardiophorine spp. and Agriotine spp.* | Northern Cyprus |  | Loggerhead (*Caretta caretta*) |  |  |  | [97, 101] |
|  | *Elater spp.* | Turkey |  | Green (*Chelonia mydas),* Loggerhead *(Caretta caretta)* | Found in sand and damaged eggs |  |  | [97] |
|  | *Lanelater sallei* | Bill Baggs Cape Florida State Park, USA |  | Loggerhead (*Caretta caretta*) | Beetle larvae eggs caused a prominent egg damage in the study (3 times worse than the damages occurred by raccoons) |  |  | [102] |
|  | *Omorgus suberosus* | Floreana Island; Quinta Playa; Galá-  pagos Islands, Ecuador; Oaxaca, Mexico |  | Green (*Chelonia mydas),* Olive ridley (*Lepidochelys olivacea*) | Predation on egg and causing mortality |  | Climate  change could also increase the impact; Rainfall increase increases the number of beetles | [103] |
|  | *Pimelia sp* | Alata beach, Dalaman beach, Dalyan İztuzu, Turkey |  | Green (*Chelonia mydas),* Loggerhead *(Caretta caretta)* | The most prominent invertebrate in the nest; may correlate with hatching failure and egg damage found in both sand and egg;  Were found mostly on top of the nest;  Occurs in nest closer to vegetation |  |  | [97, 104] |
| *Corallanidae* | *Excorallana spp.* | North-East Tobago, West Indies |  | Hawksbill (*Eretmochelys imbricata*) | Isolated from eyelid |  |  | [105] |
| *Diptera* | *Muscidae* | Dalaman, Turkey |  | Green (*Chelonia mydas*)*,* Loggerhead *(Caretta caretta)* | Less damage compared to *Pimelia spp*. And/or occurs on the egg shells opened by *Pimelia* larvae |  |  | [104] |
|  | *Platystomatidae (Duomyia foliata McAlpine and Plagiostenopterina enderleini Hendel)* | Central Queensland, Australia |  | Green (*Chelonia mydas*), Loggerhead *(Caretta caretta)* | Preferentially infest dead embryos and necrotic materials |  |  | [106] |
|  | *Sarcophagid (Eumacronychia sternali)* | Michoacán, Pacific coast of Mexico |  |  | Reducing hatching success |  |  | [101] |
|  | *Sarcophagidae (Phrosinella spp. and Eusenotainia spp.)* | Mexico; Turkey |  | Leatherback (*Dermochelys coriacea*), Olive ridley (*Lepidochelys olivacea*) | The correlation with hatching failure is not clear |  |  | [101] |
|  | *Sarcotachina aegyptiaca* | Cyprus in the eastern Mediterranean; Pacific coast of Mexico |  | Green (*Chelonia mydas*), Hawksbill (*Eretmochelys imbricata*), Loggerhead (*Caretta caretta*) | Preferentially infest dead embryos; may out-compete other species of insects. In some studies evidences found to correlate with hatching failure |  | Variation in nest temperatures, asynchronous incubation and chemical signals associated with early hatching may attract adult flies;  higher temp reduces development time | [101] |
|  | *Sarcophaga (Parasarcophaga)*  *crassipalpis, Sarcotachina subcylindric* | Mediterranean Sea |  | Green (*Chelonia mydas*), Loggerhead (*Caretta caretta*) | May be primary factors in the reduction of the success of a nest or only affect moribund eggs or embryos |  |  | [107] |
|  | *Sarcophagidae (Wohlfahrtia spp.)* | Mediterranean Sea |  | Green (*Chelonia mydas*) | May be primary factors in the reduction of the success of a nest or only affect moribund eggs or embryos |  |  | [107] |
|  | *Eumacronychia sternalis* | East Pacific; Costa Rica; Mexico |  | Green (*Chelonia mydas*), Hawksbill (*Eretmochelys imbricata*) | 30% reduction in hatching success in *Chelonia mydas* nests on the east coast of Mexico |  |  | [108] |
| *Hymenoptera* | *Formicidae (Ants)* | Rio Grande, Brazil |  | Hawksbill (*Eretmochelys imbricata*) | Attacking the hatchlings after being hatched, cases of blindness, head and flipper attack;  May attack the embryo and cause still birth;  Nests close to vegetation are more vulnerable |  |  | [109] |
|  | *Brachymeriapodagarica* | Mediterranean Sea |  | Green (*Chelonia mydas)* | May be primary factors in the reduction of  the success of a nest or only affect moribund eggs or embryos;  May exert some  Natural biological control over the sarcophagid fly infestations. |  |  | [107] |
| *Orthoptera* | *Gryllotalpidae (Scapteriscus didactylus)* | French Guiana |  | Leatherback (*Dermochelys coriacea*) | Prey on the eggs caused up to 40% damage |  |  | [110] |
| *Phoriodae* | *Megaselia scalaris* | Tortuguero, Costa Rica; Mediterranean Sea |  | Green (*Chelonia mydas*), Hawksbill (*Eretmochelys imbricata*) | Feeding on weak hatchling or dead embryos |  |  | [106, 107] |

## S7. Viruses

| Disease hazard | | Species/ region reported | Presence in sea turtles | | Outcome of infection (lesion, clinical sign and/or disease)  Symptom in individuals;  Ease of spread, rate of spread;  Diagnostic test or treatment, if available | Zoonotic/transmissible to companion animals | Correlation with climatic/anthropogenic events | Key reference(s) |
| --- | --- | --- | --- | --- | --- | --- | --- | --- |
|  |  |  | **Captive populations** | **Wild populations** |  |  |  |  |
| Herpesvirus | (*Chelonid alphaherpesvirus 5*) | Reported in tropical and sub-tropical oceans worldwide;  Found recently in new regions such as Ecuador, Malaysia and Mexico | Green (*Chelonia mydas*), Loggerhead (*Caretta caretta*) | All species: Flatback (*Natator depressus*), Green (*Chelonia mydas*), Hawksbill (*Eretmochelys imbricata),* Kemp’s ridley (*Lepidochelys kempii*), Leatherback *(Dermochelys coriacea*), Loggerhead (*Caretta caretta*), Olive ridley (*Lepidochelys olivacea*) | External and internal tumours mostly in immunocompromised individuals;  The excessive growth of tumours may be life threatening and makes the individual prone to susceptible to secondary infections and opportunistic pathogens – a likely debilitating synergy with spirorchidiasis;  Increases the risk of entanglement in monofilament line or other debris;  No significant difference in prevalence between males and females;  Transmission from a rare disease to a global threat in a short period;  Detection is based on molecular analysis and histopathology, cultured with specialised equipment and in one institute;  Surgery is conducted in some areas, in some cases after rehabilitation and release, tumour regrowth may occur and there is a varying probability of rehabilitation success reported in Southeastern parts of the USA | Not zoonotic;  Disease has spread between all sea turtles;  No evidence has been found to prove transmission to any other species | Stress and pollution seem to have a correlation with disease manifestation | [111-117] |
|  | Gray-patch disease (GPD) (Chelonid  herpesvirus 1 | Cayman Turtle Farm, Cayman Islands | Green (*Chelonia mydas*) |  | Circular popular lesion on skin that could spread and be lethal or spontaneously resolved;  May lead to secondary bacterial infection;  Control: Strict hygiene and quarantine procedures for a minimum of 3 months | No data | Overcrowding turtle tanks and higher;  Temperature may correlate with the disease | [118, 119] |
|  | lung-eye-trachea disease (LETD) (Chelonid herpesvirus 6) | Florida, USA | Green (*Chelonia mydas*) | Green *(Chelonia mydas),* Loggerhead (*Caretta caretta*) | Lesions in eye lung and trachea;  Mortality may reach 70%;  Could transmit by direct contact  Detection by molecular tools, histopathology and ELISA (antibody detection);  The first sea turtle herpes virus successfully isolated in cell culture | No data | The virus may inactivate above 30°C | [120, 121] |
|  | loggerhead genital-respiratory herpesvirus (LGVR) | Florida, USA |  | Loggerhead (*Caretta caretta*) | Respiratory and genital lesions;  Diagnosis: histopathology, molecular analysis on sample from ulcerative tissue | Possible vector: marine leeches |  | [122] |
|  | loggerhead orocutaneous herpesvirus (LOCV) | Florida, USA |  | Loggerhead (*Caretta caretta*) | Oral and cutaneous lesions;  Diagnosis: histopathology, molecular analysis on sample from ulcerative tissue | Possible vector: marine leeches |  |  |
| Papillomavirus | *Chelonia mydas* papillomavirus 1  (CmPV-1) | East Central Coast of Florida, USA |  | Loggerhead (*Caretta caretta*) | Skin lesions resolved after several months and left scars/pitted skin;  Histopathology and PCR | Species-specific |  | [123, 124] |
|  | Green (*Chelonia mydas)* papillomavirus and Loggerhead (*Caretta caretta)*  Papillomavirus isolates | North East Australia, Australia |  | Green *(Chelonia mydas),* Loggerhead (*Caretta caretta*) | Green and loggerhead skin tumours along with ChHV5;  The possible correlation with FP is under investigation |  |  | [125] |
|  | Loggerhead (*Caretta caretta)*  Papillomavirus 1 (CcPV-1) | North East Florida, USA |  | Loggerhead (*Caretta caretta*) | Skin lesion, resolved after several months;  Histopathology and PCR | Species-specific |  | [123, 124] |
| Retrovirus |  | Hawaii, USA |  | Green (*Chelonia mydas*) with FP | Incidental finding, no prove for correlation with FP disease, no correlation with any clinical disease;  Was found with laboratory test: conventional reverse transcriptase assay |  |  | [126] |
| Tornovirus | Sea turtle tornovirus 1 (STTV1) | Lake Worth Lagoon, Florida, USA |  | Green (*Chelonia mydas*) with FP | Different variations of virus have been found in infected individuals;  No correlation with FP (it hasn’t been found in most FP infected turtles) but may cause co-infections, may be commensal to turtle or an opportunistic infection |  |  | [127] |
| Betanodavirus |  | Tuscany, Italy |  | Loggerhead (*Caretta caretta*) | Isolated from eye and lung;  No evidence of pathogenic roles in sea turtles yet;  Chlorine may inactivate the virus | May infect teleost fish and cause nervous necrosis;  May infect marine invertebrates;  Sea turtles may be a carrier for this virus | Higher temperature may inactivate the virus | [20] |

## S8. Non-infectious diseases of sea turtles

|  | Health Problem | Region Reported | Species Affected | | Aetiology, if clear;  the effect on individuals, population, if known;  treatment, if stated;  mortality, morbidity if reported. | Key Reference(s) |
| --- | --- | --- | --- | --- | --- | --- |
|  |  |  | **Captive Populations** | **Wild Populations** |  |  |
| Physical Trauma | Injuries | Frequently reported | * | * | Due to predator bites, by-catch or accidents.  May happen quite often and lead to infection, minor scars and/or deep wounds. Mortality may occur if the injury is traumatic;  Appropriate modifications to vessel operation and configuration may reduce the threats;  Aggressive males may bite females during mating  Captive turtles are prone to injuries in overcrowded facilities;  Existence of rehabilitation centres in the area to surrender injured or caught turtles for healing period followed by releasing may help the population. | [128-130] |
|  | Missing organs | Frequently reported | * | * | Loss of an entire flipper or two is seen in sea turtles;  Predation and accidents may cause amputation;  Mono-filament fishing lines may tie around the flipper and lead to injuries or amputation;    Female turtles missing one or two flippers may successfully come ashore for nesting;  If the injury is fresh the creatine kinase (CK) value is high due to muscle damage. | [131] |
|  | Carapace/Plastron lesions or alterations | Canary Islands, Spain | * | * | Fracture and traumatic erosion are common and happen due to predation or accidents;  Traumatic injuries in carapace may cause lesions in lungs or kidneys;  Wound management is required according to established protocols;  Deformation and lack of ossification is also observed. | [3, 132] |
|  | Buoyancy problems | Frequently reported | * | * | The disability to submerge make individuals prone to predation, accidents and being washed ashore;  The common cause is trapped gas in intra-coelomic cavity:  In GI: due to ingestion of foreign bodies, obstructions, intussusception or constipation/obstipation.  In lung: due to pneumonia (generally pulmonary diseases), trauma leading to lung tear or internal tumours;  Spinal cord or brain trauma may also cause buoyancy disorder;  The metabolic cost of breathing is much higher for positive buoyant individuals;  If the buoyancy disorder lasts for more than a month, individuals are not allowed to be released and to avoid lung malformation external weights should be applied to normalise flotation. | [3, 133, 134] |
| Nutritional problems | Malnutrition | Frequently reported | * | * | Physical damages, high parasitic load or suffering from chronic diseases may lead to loss of appetite, nutritional deficiencies and cachexia;  Anthropological or natural habitat changes may negatively alter the nutrient intake;  Brevetoxin intoxicated individuals show low foraging activities that may lead to malnutrition;  Morphologically the animal has concave plastron, sunken eyes and muscular atrophy;  Blood test may exhibit anaemia, hypoproteinemia and hypoglycaemia;  In captivity, improper diet may cause GI obstruction and malnutrition. | [135, 136] |
|  | Metabolic bone disease |  | * | * | Low levels of calcium and high phosphorus in the diet may induce demineralization of the bones and even lead to fracture;  Gelatine-based diets, ultraviolet radiation and vitamin D3 supplements may help reduce the sign of metabolic bone disease. | [135] |
|  | Iron deficiency |  | * | Kemp’s ridley (*Lepidochelys kempii*), Loggerhead (*Caretta caretta*) | Anaemic individuals may be found lethargic, floating and hyperventilating;  In blood tests, packed cell volumes (PCVs) are lower than normal;  In captivity, diets only based on fish and squid reported to develop anaemia. | [135] |
| Environmental factors | Climate change consequences | Atlantic Ocean; Espírito Santo, Brazil; Indian Ocean; Mediterranean; North Carolina, USA; Pacific Ocean | * | Green (*Chelonia mydas*), Hawksbill (*Eretmochelys imbricata*), Kemp's ridley (*Lepidochelys kempii*), Loggerhead (*Caretta caretta*) | In case of alteration in marine biodiversity, nutrition will be affected. | [137] |
|  | Hypothermic stunning | Gulf of Mexico; Florida, New England, USA; Western Europe; Persian Gulf; Indian Lagoon | * | Green (*Chelonia mydas*), Hawksbill (*Eretmochelys imbricata*), Kemp's ridley (*Lepidochelys kempii*), Loggerhead (*Caretta caretta*) | Large numbers of stranding are reported due to cold stunning;  When individuals are exposed to low temperature water and this situation lasts for a while, it might affect them in different way (especially smaller individuals):  Water temperature = 10°C floating and not able to function, decreased heart rate, decreased circulation  Water temperature = 5-6°C shock, pneumonia, death may occur;  Salt glands functionality may decrease due to cold stunning;  Shallow waters may show more temperature fluctuations;  Rehabilitation is helpful: depending on the condition fluid therapy, antimicrobial treatment, medications, and prolonged care maybe required. | [138, 139] |
| Anthropogenic problems | Entanglements |  | * | * | Entanglement in man-made objects such as fishing gears may cause minor and major injuries;  If the entanglement keeps individuals submerged for a while anoxia may occur;  Due to interactional behaviour, flipper entanglement is more probable;  In pelagic stages, entanglement in buoyant objects is happening frequently. | [140, 141] |
|  | Accidents and injuries | * | * | * | Collision with maritime traffic: fishing vessels, (oil and cargo) tankers, beach leisure facilities; happens in different regions;  It may cause mortality or injuries (minor or major) (See ‘Injuries’ in ‘*Physical Problems’* column above). | [142] |
|  | By-catch injuries | Frequently reported | Not applicable | All species | Trawl and grill net and fishing lines may keep individuals submerged for a long time and put individuals at risk of drowning or an anoxic state;  Loggerheads (*Caretta caretta*) interact with more fisheries than any other species. One individual was reported injured by an eagle ray spine in a trawl net in Florida, USA;  These methods may help reducing the risk: turtle exclusion devices (TEDs); larger round shape hooks; using fish bait; avoiding bycatch hotspots; setting the gears deeper, reducing the soaking time and retrieving during the day. | [128, 141, 143] |
|  | Debris ingestion | * | * | Green (*Chelonia mydas*), Kemp’s ridley (*Lepidochelys kempii),* Leatherback (*Dermochelys coriacea*) | Individuals ingest debris frequently and these foreign objects may affect the health condition;  Young individuals in pelagic stages are at higher risk of eating or entangling in buoyant wastes;  Plastic is one of the main debris, tar and crude oil are the other examples;  Debris may block the GI and accumulate intestinal gas; cause local ulcerations; interfere with metabolism; be toxic for the body;  Gelatinivorous species such as Leatherbacks (*Dermochelys coriacea*) are more likely to ingest debris, and Kemp’s ridley are less likely to ingest debris. | [140, 144, 145] |
|  | Problems with chemical and organic pollutant | Reported globally | * | All species | Chemical pollutants are not biodegradable and normally persist in the release site. Such pollutants like oil may cause fouling, entanglement, external necrosis and skin sloughing, it may also interfere with salt gland functionality;  Agricultural waste may elevate the nutrient level in the ocean and stimulate harmful algal and cyanobacterial blooms. This incidence may directly harm individuals (stranding and mortality is also reported) or correlate with other diseases such as FP;  High concentration of PCBs, OC insecticides, persistent organic pollutants, algal toxins, metals and trace elements may cause a variety of adverse health effects such as immunosuppression /immunostimulation;  Immunosuppressive cofactors are likely to correlate with FP. | [136, 144, 146, 147] |
| Medical problem | Neurological disease |  | * | Loggerhead (*Caretta caretta*) Green (*Chelonia mydas*), Kemp's ridley (*Lepidochelys kempii)* | Diagnosis is not easy but neurological signs may be atypical behaviours such as circling or non-responsiveness, head bobbing, muscle twitching, and jerky body movements;  Neurologic spirorchiidiasis is reported in loggerheads. Normally diagnosis is postmortem;  Brevetoxin produced by dinoflagellate may cause neurological signs. Stranding and mortality are also reported;  Dehydration, systemic antihistamine treatment and supportive care seems to be helpful in this situation;  Pollutant metals may induce lesions in the central nervous system. | [146, 148-150] |
|  | Pulmonary disease | Frequently reported | * | * | Pneumonia is a common pulmonary disease; the aetiology may be infectious or non-infectious;  Traumatic injuries in the carapace may also lead to pulmonary lesions;  Pulmonary diseases may lead to buoyancy disorders. | [3] |
|  | Debilitated Turtle Syndrome |  | * | Green (*Chelonia mydas*)*,* Loggerhead (*Caretta caretta),*  Olive ridley (*Lepidochelys olivacea*) | The cause is unknown, cold stunning may be an initial cause;  End stage disease with following signs: emaciated, lethargic, hypoglycaemic, anaemic, and heavily covered with epibiota  Secondary infections may also occur – individuals may be immunosuppressed;  Rehabilitation may be helpful: fluid therapy, treating anaemia, hyperglycaemia, antimicrobial treatments, nutritional supports, medical treatments due to diagnosed internal problems. | [151-153] |

* There is not enough information about the species or the region

## References

1. Hartley M, Sainsbury A. Methods of disease risk analysis in wildlife translocations for conservation purposes. EcoHealth. 2017;14(1):16-29.

2. Jakob-Hoff RM, MacDiarmid SC, Lees C, Miller PS, Travis D, Kock R. original: Manual of Procedures for Wildlife Disease Risk Analysis. Co-published by OIE and IUCN2014.

3. Orós J, Torrent A, Calabuig P, Déniz S. Diseases and causes of mortality among sea turtles stranded in the Canary Islands, Spain (1998–2001). Diseases of aquatic organisms. 2005;63(1):13-24.

4. Candan O, Candan ED. Bacterial diversity of the green turtle (Chelonia mydas) nest environment. Science of the Total Environment. 2020;720. doi: 10.1016/j.scitotenv.2020.137717.

5. Aguirre AA. Occurrence of potential pathogens in green sea turtles (Chelonia mydas) afflicted or free of fibropapillomas in Kaneohe Bay, Island of Oahu, Hawaii, 1991. United States: University of California Libraries; 1992.

6. Aguirre AA, Balazs GH, Zimmerman B, Spraker TR. Evaluation of Hawaiian green turtles (Chelonia mydas) for potential pathogens associated with fibropapillomas. Journal of Wildlife Diseases. 1994;30(1):8-15.

7. Campbell RS, Glazebrook JS. A survey of the diseases of marine turtles in northern Australia I : farmed turtles. 1990.

8. Glazebrook J, Campbell R, Thomas A. Studies on an ulcerative stomatitis, obstructive rhinitis pneumonia disease complex in hatchling and juvenile sea turtles Chelonia mydas and Caretta caretta. Diseases of Aquatic Organisms. 1993;16(2):133-47.

9. Orós J, Camacho M, Calabuig P, Arencibia A. Salt gland adenitis as only cause of stranding of loggerhead sea turtles Caretta caretta. Diseases of aquatic organisms. 2011;95(2):163-6.

10. Work TM, Balazs GH, Wolcott M, Morris R. Bacteraemia in free-ranging Hawaiian green turtles Chelonia mydas with fibropapillomatosis. Diseases of aquatic organisms. 2003;53(1):41-6.

11. Buller NB. Bacteria and Fungi from Fish and other Aquatic Animals: a practical identification manual: Cabi; 2014.

12. Awong-Taylor J, Craven KS, Griffiths L, Bass C, Muscarella M. Comparison of biochemical and molecular methods for the identification of bacterial isolates associated with failed loggerhead sea turtle eggs. J Appl Microbiol. 2008;104(5):1244-51. Epub 2007/11/22. doi: 10.1111/j.1365-2672.2007.03650.x. PubMed PMID: 18028359.

13. Chuen-Im T, Areekijserre M, Chongthammakun S, Graham SV. Aerobic Bacterial Infections in Captive Juvenile Green Turtles (Chelonia mydas) and Hawksbill Turtles (Eretmochelys imbricata) from Thailand. Chelonian Conservation and Biology. 2010;9(1):135-42. PubMed PMID: 612772579.

14. Chinnadurai SK, Devoe RS. Selected infectious diseases of reptiles. Vet Clin North Am Exot Anim Pract. 2009;12(3):583-96, Table of Contents. Epub 2009/09/08. doi: 10.1016/j.cvex.2009.06.008. PubMed PMID: 19732710.

15. Raidal SR, Ohara M, Hobbs RP, Prince RIT. Gram-negative bacterial infections and cardiovascular parasitism in green sea turtles (Chelonia mydas). Australian Veterinary Journal. 1998;76(6):415-7. doi: 10.1111/j.1751-0813.1998.tb12392.x.

16. Foti; M, Bottari; T, Coci; G, Daidone; A, Maria Grazia Pennisi. Enterobacteriaceae Isolates in Cloacal Swabs from Live-stranded Internally-hooked Loggerhead Sea Turtles, Caretta caretta, in the Central Mediterranean Sea. Journal of Herpetological Medicine and Surgery. 2008;17(4).

17. Foti M, Giacopello C, Bottari T, Fisichella V, Rinaldo D, Mammina C. Antibiotic Resistance of Gram Negatives isolates from loggerhead sea turtles (Caretta caretta) in the central Mediterranean Sea. Mar Pollut Bull. 2009;58(9):1363-6. Epub 2009/05/29. doi: 10.1016/j.marpolbul.2009.04.020. PubMed PMID: 19473669.

18. Keene E, Soule T, Paladino F. Microbial Isolations from Olive Ridley (Lepidochelys olivacea) and East Pacific Green (Chelonia mydas agassizii) Sea Turtle Nests in Pacific Costa Rica, and Testing of Cloacal Fluid Antimicrobial Properties. Chelonian Conservation and Biology. 2014;13(1):49-55. PubMed PMID: 1551140132.

19. Zieger U, Trelease H, Winkler N, Mathew V, Sharma RN. Bacterial Contamination of Leatherback Turtle (Dermochelys coriacea) eggs and sand in nesting chambers at Levera Beach, Grenada, West Indies-a preliminary study. West indian veterinary journal. 2009;9(2):21-6.

20. Fichi G, Cardeti G, Cersini A, Mancusi C, Guarducci M, Di Guardo G, et al. Bacterial and viral pathogens detected in sea turtles stranded along the coast of Tuscany, Italy. Veterinary Microbiology. 2016;185:56-61. doi: http://dx.doi.org/10.1016/j.vetmic.2016.02.003.

21. Al-Bahry SN, Al-Zadjali MA, Mahmoud IY, Elshafie AE. Biomonitoring marine habitats in reference to antibiotic resistant bacteria and ampicillin resistance determinants from oviductal fluid of the nesting green sea turtle, Chelonia mydas. Chemosphere. 2012;87(11):1308-15. doi: http://dx.doi.org/10.1016/j.chemosphere.2012.01.051.

22. George RH. Health problems and diseases of sea turtles. In: Lutz PL, Musick JA, editors. The Biology of Sea Turtle. 1. Florida: CRC Press; 1997.

23. Wyneken J, Burke TJ, Salmon M, Pedersen DK. Egg Failure in Natural and Relocated Sea Turtle Nests. Journal of Herpetology. 1988;22(1):88-96. doi: 10.2307/1564360.

24. Santoro M, Hernández G, Caballero M, García F. AEROBIC BACTERIAL FLORA OF NESTING GREEN TURTLES (CHELONIA MYDAS) FROM TORTUGUERO NATIONAL PARK, COSTA RICA. Journal of Zoo and Wildlife Medicine. 2006;37(4):549-52. doi: 10.1638/05-118.1.

25. Zavala-Norzagaray AA, Aguirre AA, Velazquez-Roman J, Flores-Villaseñor H, León-Sicairos N, Ley-Quiñonez CP, et al. Isolation, characterization, and antibiotic resistance of Vibrio spp. in sea turtles from Northwestern Mexico. Frontiers in Microbiology. 2015;6:635. doi: 10.3389/fmicb.2015.00635. PubMed PMID: PMC4480150.

26. Aguirre AA, Gardner SC, Marsh JC, Delgado SG, Limpus CJ, Nichols WJ. Hazards Associated with the Consumption of Sea Turtle Meat and Eggs: A Review for Health Care Workers and the General Public. EcoHealth. 2006;3(3):141-53. doi: 10.1007/s10393-006-0032-x.

27. Oros J, Calabuig P, Deniz S. Digestive pathology of sea turtles stranded in the Canary Islands between 1993 and 2001. Vet Rec. 2004;155(6):169-74. Epub 2004/09/11. PubMed PMID: 15357377.

28. Obendorf D, Carson J, McManus T. Vibrio damsela infection in a stranded leatherback turtle (Dermochelys coriacea). Journal of wildlife diseases. 1987;23(4):666-8.

29. Alfaro A, Køie M, Buchmann K, editors. Synopsis of infections in sea turtles caused by virus, bacteria and parasites: an ecological review. 27th Annual Symposium on Sea Turtle Biology and Conservation; 2008: NOAA Tech Memo.

30. O'Grady KA, Krause V. An outbreak of salmonellosis linked to a marine turtle. Southeast Asian J Trop Med Public Health. 1999;30(2):324-7. Epub 2000/04/25. PubMed PMID: 10774704.

31. Dutton CS, Revan F, Wang C, Xu C, Norton TM, Stewart KM, et al. SALMONELLA ENTERICA PREVALENCE IN LEATHERBACK SEA TURTLES (DERMOCHELYS CORIACEA) IN ST. KITTS, WEST INDIES. Journal of Zoo and Wildlife Medicine. 2013;44(3):765-8.

32. Work TM, Dagenais J, Stacy BA, Ladner JT, Lorch JM, Balazs GH, et al. A novel host-adapted strain of Salmonella Typhimurium causes renal disease in olive ridley turtles (Lepidochelys olivacea) in the Pacific. Scientific reports. 2019;9(1):1-13.

33. Oros J, Calabuig P, Arencibia A, Camacho M, Jensen H. Systemic mycosis caused by Trichophyton spp. in an olive ridley sea turtle (Lepidochelys olivacea): an immunohistochemical study. N Z Vet J. 2011;59(2):92-5. Epub 2011/03/17. doi: 10.1080/00480169.2011.552859. PubMed PMID: 21409736.

34. Oros J, Arencibia A, Fernandez L, Jensen HE. Intestinal candidiasis in a loggerhead sea turtle (Caretta caretta): an immunohistochemical study. Vet J. 2004;167(2):202-7. Epub 2004/02/21. doi: 10.1016/s1090-0233(03)00111-4. PubMed PMID: 14975396.

35. IUCN. The IUCN Red List of Threatened Species 2018. Available from: http://www.iucnredlist.org/.

36. Lu HK, Chen EF, Xie SY, Chai CL, Wei YD, Mo ST, et al. [Investigation on vibrio cholera carried in aquatic products of littoral areas, Zhejiang Province]. Zhonghua Yu Fang Yi Xue Za Zhi. 2006;40(5):336-8. Epub 2006/12/15. PubMed PMID: 17166425.

37. Acuña MT, Díaz G, Bolaños H, Barquero C, Sánchez O, Sánchez LM, et al. Sources of Vibrio mimicus Contamination of Turtle Eggs. Applied and Environmental Microbiology. 1999;65(1):336-8. PubMed PMID: PMC91027.

38. Guthrie A, George J, deMaar TW. Bilateral Chronic Shoulder Infections in an Adult Green Sea Turtle (Chelonia mydas). Journal of Herpetological Medicine and Surgery. 2010;20(4):105-8. doi: 10.5818/1529-9651-20.4.105.

39. Innis CJ, Braverman H, Cavin JM, Ceresia ML, Baden LR, Kuhn DM, et al. Diagnosis and management of Enterococcus spp infections during rehabilitation of cold-stunned Kemp's ridley turtles (Lepidochelys kempii): 50 cases (2006–2012). Journal of the American Veterinary Medical Association. 2014;245(3):315-23.

40. Torrent A, Deniz S, Ruiz A, Calabuig P, Sicilia J, Oros J. Esophageal diverticulum associated with Aerococcus viridans infection in a loggerhead sea turtle (Caretta caretta). J Wildl Dis. 2002;38(1):221-3. Epub 2002/02/13. doi: 10.7589/0090-3558-38.1.221. PubMed PMID: 11838222.

41. Keene EL. Microorganisms from sand, cloacal fluid, and eggs of Lepidochelys olivacea and standard testing of cloacal fluid antimicrobial properties. department of biology: Indiana University-Purdue University Fort Wayne; 2012.

42. Homer BL, Jacobson ER, Schumacher J, Scherba G. Chlamydiosis in Mariculture-reared Green Sea Turtles (Chelonia mydas). Vet Pathol. 1994;31(1).

43. Arena PC, Warwick C, Steedman C. Welfare and Environmental Implications of Farmed Sea Turtles. Journal of Agricultural and Environmental Ethics. 2014;27(2):309-30. doi: 10.1007/s10806-013-9465-8.

44. Brock JA, Nakamura RM, Miyahara AY, Chang EML. Tuberculosis in Pacific Green Sea Turtles, Chelonia mydas. Transactions of the American Fisheries Society. 1976;105(4):564-6. doi: 10.1577/1548-8659(1976)105<564:TIPGST>2.0.CO;2.

45. Greer LL, Strandberg JD, Whitaker BR. Mycobacterium chelonae osteoarthritis in a Kemp's ridley sea turtle (Lepidochelys kempii). J Wildl Dis. 2003;39(3):736-41. Epub 2003/10/22. doi: 10.7589/0090-3558-39.3.736. PubMed PMID: 14567241.

46. Nardini G, Florio D, Girolamo ND, Gustinelli A, Quaglio F, Fiorentini L, et al. DISSEMINATED MYCOBACTERIOSIS IN A STRANDED LOGGERHEAD SEA TURTLE (CARETTA CARETTA). Journal of Zoo and Wildlife Medicine. 2014;45(2):357-60. doi: doi:10.1638/2013-0252R1.1.

47. Donnelly K, Waltzek TB, Wellehan JF, Jr., Stacy NI, Chadam M, Stacy BA. Mycobacterium haemophilum infection in a juvenile leatherback sea turtle (Dermochelys coriacea). J Vet Diagn Invest. 2016;28(6):718-21. Epub 2016/10/05. doi: 10.1177/1040638716661746. PubMed PMID: 27698171.

48. Leong JK, Smith DL, Revera DB, Lewis DH, Scott JL, DiNuzzo AR, editors. Health care and diseases of captive-reared loggerhead and Kemp’s ridley sea turtles. Proceedings of the First International Symposium on Kemp’s Ridley Sea Turtle Biology, Conservation and Management; 1989; Texas A&M University, Sea Grant College Program, College Station, TX.

49. Herbst LH, Jacobson ER. Practical approaches for studying sea turtle health and disease. In: Lutz L, Musick JA, editors. The Biology of Sea Turtles. 2. Florida, United States: CRC Press; 2002. p. 385-410.

50. Phillott AD, Parmenter CJ, Limpus CJ, Harrower K. Mycobiota as acute and chronic cloacal contaminants of female sea turtles. Australian journal of zoology. 2002;50(6):687-95.

51. Rédou V, Navarri M, Meslet-Cladière L, Barbier G, Burgaud G. Species Richness and Adaptation of Marine Fungi from Deep-Subseafloor Sediments. Applied and Environmental Microbiology. 2015;81(10):3571-83. doi: 10.1128/AEM.04064-14. PubMed PMID: PMC4407237.

52. Mo CL, Salas I, M C, editors. Are fungi and bacteria responsible for olive ridley's egg lost? Tenth Annual Workshop on Sea Turtle Biology and Conservation; 1990; Hilton Head Island, South Carolina: NOAA Technical Memorandum NMFS-SEFC-278.

53. Domiciano IG, Domit C, Trigo CC, de Alcântara BK, Headley SA, Bracarense APF. Phaeohyphomycoses in a Free-Ranging Loggerhead Turtle (Caretta caretta) from Southern Brazil. Mycopathologia. 2014;178(1-2):123-8.

54. Phillott AD, Elsmore S. Black noddies (Anous minutus) and wedge-tailed shearwaters (Puffinus pacificus) as potential hosts for fungi invading sea turtle nests at Heron Island, Queensland. Transactions of the Royal Society of South Australia. 2004;128:73-.

55. Phillott AD, Parmenter CJ, Limpus CJ. Occurrence of mycobiota in eastern Australian sea turtle nests. Memoirs of the Queensland Museum. 2004;49:701-3.

56. Güçlü Ö, Bıyık H, Şahiner A. Mycoflora identified from loggerhead turtle (Caretta caretta) egg shells and nest sand at Fethiye beach, Turkey. African Journal of Microbiology Research. 2010;4(5):408-13.

57. Smith KA. Nitrous oxide and climate change: Earthscan; 2010.

58. Woo PCY, Leung S-Y, Ngan AHY, Lau SKP, Yuen K-Y. A significant number of reported Absidia corymbifera (Lichtheimia corymbifera) infections are caused by Lichtheimia ramosa (syn. Lichtheimia hongkongensis): an emerging cause of mucormycosis. Emerg Microbes Infect. 2012;1:e15.

59. Jacobson E, Gaskin J, Shields R, White F. Mycotic pneumonia in mariculture-reared green sea turtles. Journal of the American Veterinary Medical Association. 1979;175(9):929-33.

60. Manire CA, Rhinehart HL, Sutton DA, Thompson EH, Rinaldi MG, Buck JD, et al. Disseminated mycotic infection caused by Colletotrichum acutatum in a Kemp's ridley sea turtle (Lepidochelys kempi). Journal of clinical microbiology. 2002;40(11):4273-80.

61. Allender MC, Dreslik M, Wylie S, Phillips C, Wylie DB, Maddox C, et al. Chrysosporium sp. Infection in Eastern Massasauga Rattlesnakes. Emerging Infectious Diseases. 2011;17(12):2383-4. doi: 10.3201/eid1712.110240. PubMed PMID: PMC3311193.

62. Sison T, Padilla M, Vizmanos M, Follosco M. Isolation and identification of fungi found in necrotic skin lesions of captive marine turtles (Eretmochelys imbricata). Philippine Journal of Veterinary Medicine. 1990;27(2):35-6.

63. Oros J, Delgado C, Fernandez L, Jensen HE. Pulmonary hyalohyphomycosis caused by Fusarium spp in a Kemp's ridley sea turtle (Lepidochelys kempi): an immunohistochemical study. N Z Vet J. 2004;52(3):150-2. Epub 2005/03/16. doi: 10.1080/00480169.2004.36420. PubMed PMID: 15768112.

64. Sarmiento-Ramírez JM, Abella-Pérez E, Phillott AD, Sim J, van West P, Martín MP, et al. Global Distribution of Two Fungal Pathogens Threatening Endangered Sea Turtles. PLoS ONE. 2014;9(1):e85853. doi: 10.1371/journal.pone.0085853.

65. Cafarchia C, Paradies R, Figueredo LA, Iatta R, Desantis S, Di Bello AVF, et al. Fusarium spp. in Loggerhead Sea Turtles (Caretta caretta): From Colonization to Infection. Veterinary Pathology. 2020;57(1):139-46. doi: 10.1177/0300985819880347.

66. Cabanes F, Alonso J, Castella G, Alegre F, Domingo M, Pont S. Cutaneous hyalohyphomycosis caused by Fusarium solani in a loggerhead sea turtle (Caretta caretta L.). Journal of clinical microbiology. 1997;35(12):3343-5.

67. Sarmiento-Ramírez JM, Abella E, Martín MP, Tellería MT, López-Jurado LF, Marco A, et al. Fusarium solani is responsible for mass mortalities in nests of loggerhead sea turtle, Caretta caretta, in Boavista, Cape Verde. FEMS Microbiology Letters. 2010;312(2):192-200. doi: 10.1111/j.1574-6968.2010.02116.x.

68. Posthaus H, Krampe M, Pagan O, Gueho E, Suter C, Bacciarini L. Systemic paecilomycosis in a hawksbill turtle (Eretmochelys imbricata). Journal de mycologie médicale. 1997;7(4):223-6.

69. Schumacher VL, Mangold B, Lenzycki J, Hinckley L, Sutton DA, Frasca S. Occurrence of fruiting structures allows determination of Purpureocillium lilacinum as an inciting agent of pleuritis and pneumonia in a loggerhead sea turtle (Caretta caretta) by histopathologic correlation to culture. Medical Mycology Case Reports. 2014;6:42-5. doi: 10.1016/j.mmcr.2014.07.007. PubMed PMID: PMC4216333.

70. Arpini CM, Nóbrega YC, Castheloge VD, Neves DS, Tadokoro CE, Costa GLD, et al. Purpuriocillium lilacinum infection in captive loggerhead sea turtle hatchlings. Medical Mycology Case Reports. 2019;23:8-11. doi: 10.1016/j.mmcr.2018.10.002.

71. Bailey T. Mortality at a Hawksbill turtle (Eretmochelys Imbricata) rearing center. Wildlife Middle East News 2008.

72. Gordon AN. A necropsy-based study of green turtles (Chelonia mydas) in South-East Queensland. 2005.

73. Chapman PA, Owen H, Flint M, Traub RJ, Cribb TH, Mills PC. Molecular Characterization of Coccidia Associated with an Epizootic in Green Sea Turtles (Chelonia mydas) in South East Queensland, Australia. PLoS ONE. 2016;11(2):e0149962. doi: 10.1371/journal.pone.0149962. PubMed PMID: PMC4763108.

74. Graczyk TK, Balazs GH, Work T, Aguirre AA, Ellis DM, Murakawa S, et al. Cryptosporidium sp. Infections in Green Turtles, Chelonia mydas, as a Potential Source of Marine Waterborne Oocysts in the Hawaiian Islands. Applied and Environmental Microbiology. 1997;63(7):2925-7.

75. Upton SJ, Odell DK, Walsh MT. Eimeria caretta sp. nov.(Apicomplexa: Eimeriidae) from the loggerhead sea turtle, Caretta caretta (Testudines). Canadian journal of zoology. 1990;68(6):1268-9.

76. Santoro M, Mattiucci S, Paoletti M, Liotta A, Degli Uberti B, Galiero G, et al. Molecular identification and pathology of Anisakis pegreffii (Nematoda: Anisakidae) infection in the Mediterranean loggerhead sea turtle (Caretta caretta). Veterinary parasitology. 2010;174(1):65-71.

77. Werneck MR, Gallo BMG, Silva RJ. Spirorchiids (Digenea : Spirorchiidae) infecting a Hawksbill sea turtle Eretmochelys imbricata (Linnaeus 1758) from Brazil. Arquivo Brasileiro De Medicina Veterinaria E Zootecnia. 2008;60(3):663-6. doi: 10.1590/s0102-09352008000300021. PubMed PMID: WOS:000257899200021.

78. Santoro M, Marchiori E, Palomba M, Uberti BD, Marcer F, Mattiucci S. The mediterranean mussel (Mytilus galloprovincialis) as intermediate host for the anisakid sulcascaris sulcata (Nematoda), a pathogen parasite of the mediterranean loggerhead turtle (caretta caretta). Pathogens. 2020;9(2). doi: 10.3390/pathogens9020118.

79. Marcer F, Tosi F, Franzo G, Vetri A, Ravagnan S, Santoro M, et al. Updates on Ecology and Life Cycle of Sulcascaris sulcata (Nematoda: Anisakidae) in Mediterranean Grounds: Molecular Identification of Larvae Infecting Edible Scallops. Frontiers in Veterinary Science. 2020;7. doi: 10.3389/fvets.2020.00064.

80. Aznar FJ, Badillo FJ, Raga JA. Gastrointestinal Helminths of Loggerhead Turtles (Caretta caretta) from the Western Mediterranean: Constraints on Community Structure. The Journal of Parasitology. 1998;84(3):474-9. doi: 10.2307/3284708.

81. Gracan R, Buršic M, Mladineo I, Kucinic M, Lazar B, Lackovic G. Gastrointestinal helminth community of loggerhead sea turtle Caretta caretta in the Adriatic Sea. Diseases of aquatic organisms. 2012;99(3):227.

82. Lester R, Blair D, Heald D. Nematodes from scallops and turtles from Shark Bay, Westren Australia. Marine and Freshwater Research. 1980;31(5):713-7.

83. Santoro M, Mattiucci S. Sea Turtle Parasites. In: Wehrtmann IS, Cortés J, editors. Marine Biodiversity of Costa Rica, Central America. Dordrecht: Springer Netherlands; 2009. p. 507-19.

84. Santoro M, Greiner EC, Morales JA, Rodriguez-Ortiz B. Digenetic trematode community in nesting green sea turtles (Chelonia mydas) from Tortuguero National Park, Costa Rica. J Parasitol. 2006;92(6):1202-6. Epub 2007/02/20. doi: 10.1645/ge-866r.1. PubMed PMID: 17304795.

85. Werneck M, Lima E, Pires T, Silva R. Helminth Parasites of the Juvenile Hawksbill Turtle Eretmochelys imbricata (Testudines: Cheloniidae) in Brazil. The Journal of parasitology. 2015;101(4):500-3.

86. Marangi M, Carlino P, Profico C, Olivieri V, Totaro G, Furii G, et al. First multicenter coprological survey on helminth parasite communities of free-living loggerhead sea turtles Caretta caretta (Linnaeus, 1758) from the Adriatic Sea and Northern Ionian Sea. International Journal for Parasitology: Parasites and Wildlife. 2020;11:207-12. doi: 10.1016/j.ijppaw.2020.02.006.

87. Valente AL, Parga ML, Espada Y, Lavin S, Alegre F, Marco I, et al. Evaluation of Doppler ultrasonography for the measurement of blood flow in young loggerhead sea turtles (Caretta caretta). The Veterinary Journal. 2008;176(3):385-92.

88. Santoro M, Badillo FJ, Mattiucci S, Nascetti G, Bentivegna F, Insacco G, et al. Helminth communities of loggerhead turtles (Caretta caretta) from Central and Western Mediterranean Sea: The importance of host's ontogeny. Parasitology International. 2010;59(3):367-75.

89. Gomes M, Martins I, Werneck M, Pavanelli L. Community ecology of gastrointestinal helminths from green turtles (Chelonia mydas) collected in the coast of Espírito Santo. Arquivo Brasileiro de Medicina Veterinária e Zootecnia. 2017;69(3):644-50.

90. Wolke RE, Brooks DR, George A. SPIRORCHIDIASIS IN LOGGERHEAD SEA TURTLES (CARETTA CARETTA): PATHOLOGY. Journal of Wildlife Diseases. 1982;18(2):175-85. doi: 10.7589/0090-3558-18.2.175.

91. Flint M, Eden PA, Limpus CJ, Owen H, Gaus C, Mills PC. Clinical and Pathological Findings in Green Turtles (Chelonia mydas) from Gladstone, Queensland: Investigations of a Stranding Epidemic. EcoHealth. 2015;12(2):298-309. doi: 10.1007/s10393-014-0972-5.

92. Graczyk TK, Aguirre AA, Balazs GH. Detection by ELISA of Circulating Anti-Blood Fluke (Carettacola, Hapalotrema, and Learedius) Immunoglobulins in Hawaiian Green Turtles (Chelonia mydas). The Journal of Parasitology. 1995;81(3):416-21. doi: 10.2307/3283824.

93. Chen H, Kuo R, Chang T, Hus C, Bray R, Cheng I. Fluke (Spirorchiidae) infections in sea turtles stranded on Taiwan: prevalence and pathology. Journal of Parasitology. 2012;98(2):437-9.

94. Werneck M, Nunes C, Jerdy H, Carvalho E. Loggerhead turtle, Caretta caretta (Linnaeus, 1758)(Testudines, Cheloniidae), as a new host of Monticellius indicum Mehra, 1939 (Digenea: Spirorchiidae) and associated lesiond to spirorchiid eggs. Helminthologia. 2017;54(4):363-8.

95. Blair D. A Revision of the Subfamily Octangiinae (Platyhelminthes, Digenea, Microscaphidiidae) Parasitic in Marine Turtles (Reptilia, Chelonia). Australian journal of zoology. 1987;35(1):75-92.

96. Werneck M, Silva R. Styphlotrema solitaria Looss, 1899 (Digenea, Styphlotrematidae) infecting Eretmochelys imbricata Linnaeus 1758 (Testudines, Chelonidae) in Brazil. Neotropical Helminthology. 2012;6(1):121-6.

97. Cemil AYMAK, Serap Ergene GOZUKARA, Yusuf KATILMIS, Rasit URHAN, UCAR AH, editors. Invertebrate infestation in Green Turtle Chelonia mydas and Loggerhead Turtle Caretta caretta nests, in Alata Beach, Mersin, Turkey. Proceedings, Second Mediterranean Conference on Marine Turtles Barcelona Convention – Bern Convention – Bonn Convention; 2005; Kemer, Antalya, Turkey.

98. Rodenbusch CR, Marks FS, Canal CW, Reck J. Marine leech Ozobranchus margoi parasitizing loggerhead turtle (Caretta caretta) in Rio Grande do Sul, Brazil. Revista Brasileira de Parasitologia Veterinária. 2012;21(3):301-3.

99. Glazebrook JS, Campbell RSF. A SURVEY OF THE DISEASES OF MARINE TURTLES IN NORTHERN AUSTRALIA .2. OCEANARIUM-REARED AND WILD TURTLES. DISEASES OF AQUATIC ORGANISMS. 1990;9(2):97-104.

100. Vivaldo SG, Sarabia DO, Salazar CP, Hernández ÁG, Lezama JR. Identificación de parásitos y epibiontes de la tortuga Golfina (Lepidochelys olivacea) que arribó a playas de Michoacán y Oaxaca, México Identification of parasites and epibionts in the Olive Ridley Turtle (Lepidochelys olivacea) that arrived to the beaches. Vet Méx. 2006;37:4.

101. McGowan A, Broderick A, Deeming J, Godley B, Hancock E. Dipteran infestation of loggerhead (Caretta caretta) and green (Chelonia mydas) sea turtle nests in northern Cyprus. Journal of Natural History. 2001;35(4):573-81.

102. Donlan EM, Townsend JH, Golden EA. Predation of Caretta caretta (Testudines: Cheloniidae) eggs by larvae of Lanelater sallei (Coleoptera: Elateridae) on Key Biscayne, Florida. Caribbean Journal of Science. 2004;40:415-20.

103. Zárate P, Bjorndal KA, Parra M, Dutton PH, Seminoff JA, Bolten AB. Hatching and emergence success in green turtle Chelonia mydas nests in the Galápagos Islands. Aquatic Biology. 2013;19(3):217-29.

104. Katılmış Y, Urhan R, Kaska Y, Başkale E. Invertebrate infestation on eggs and hatchlings of the loggerhead turtle, Caretta caretta, in Dalaman, Turkey. In: Hawksworth DL, Bull AT, editors. Marine, Freshwater, and Wetlands Biodiversity Conservation. Dordrecht: Springer Netherlands; 2006. p. 353-62.

105. walker G, M W, H H, R D, editors. Parasites of Hawksbill turtles, Eretmochelys imbricata, nesting in north-east Tobago, West Indies. 38th Annual Symposium on Sea Turtle Biology and Conservation; 2017; Kobe, Japan.

106. Hall SCB, Parmenter CJ. Larvae of two signal fly species (Diptera:Platystomatidae), Duomyia foliata McAlpine and Plagiostenopterina enderleini Hendel, are scavengers of sea turtle eggs. Australian Journal of Zoology. 2006;24(4):245-52. doi: 10.1071/ZO06025.

107. Broderick AC, Hancock EG. Insect infestation of Mediterranean marine turtle eggs. Herpetological Review. 1997;28(4):190-1.

108. Lopes HdS. On Eumacronychia sternalis Allen (Diptera, Sarcophagidae) with larvae living on eggs and hatchlings of the east Pacific green turtle. Revista Brasileira de Biologia. 1982.

109. da Silva PF, Chaves MF, Santos MG, Santos AJB, Magalhães MdS, Andreazze R, et al. Insect Infestation of Hawksbill Sea Turtle Eggs in Rio Grande do Norte, Brazil. Chelonian Conservation and Biology. 2016;15(1):147-53. doi: 10.2744/CCB-1133.1.

110. Maros A, Louveaux A, Godfrey MH, Girondot M. Scapteriscus didactylus (Orthoptera, Gryllotalpidae), predator of leatherback turtle eggs in French Guiana. Marine Ecology Progress Series. 2003;249:289-96.

111. Herbst L, Eckert K, Bjorndal K, Abreu-Grobois F, Donnelly M. Infectious diseases of marine turtles. Research and management techniques for the conservation of sea turtles IUCN/SSC Marine Turtle Specialist Group, Washington, DC. 1999.

112. Lackovich JK, Brown DR, Homer BL, Garber RL, Mader DR, Moretti RH, et al. Association of herpesvirus with fibropapillomatosis of the green turtle Chelonia mydas and the loggerhead turtle Caretta caretta in Florida. Diseases of aquatic organisms. 1999;37(2):89-97.

113. Work TM, Balazs GH, Rameyer RA, Morris RA. Retrospective pathology survey of green turtles Chelonia mydas with fibropapillomatosis in the Hawaiian Islands, 1993-2003. Diseases of aquatic organisms. 2004;62(1):163-76.

114. Jones K, Ariel E, Burgess G, Read M. A review of fibropapillomatosis in Green turtles (Chelonia mydas). The Veterinary Journal. 2016;212:48-57. doi: http://dx.doi.org/10.1016/j.tvjl.2015.10.041.

115. Work TM, Dagenais J, Weatherby TM, Balazs GH, Ackermann M. In Vitro Replication of Chelonid Herpesvirus 5 in Organotypic Skin Cultures from Hawaiian Green Turtles (Chelonia mydas). J Virol. 2017;91(17). Epub 2017/06/16. doi: 10.1128/jvi.00404-17. PubMed PMID: 28615209; PubMed Central PMCID: PMCPMC5553171.

116. Cárdenas DM, Cucalón RV, Medina-Magües LG, Jones K, Alemán RA, Alfaro-Núñez A, et al. Fibropapillomatosis in a Green Sea Turtle (Chelonia mydas) from the Southeastern Pacific. Journal of wildlife diseases. 2019;55(1):169-73.

117. Page-Karjian A, Perrault JR, Zirkelbach B, Pescatore J, Riley R, Stadler M, et al. Tumor re-growth, case outcome, and tumor scoring systems in rehabilitated green turtles with fibropapillomatosis. Diseases of Aquatic Organisms. 2019;137(2):101-8. doi: 10.3354/dao03426.

118. Rebell G, Rywlin A, Haines H. A herpesvirus-type agent associated with skin lesions of green sea turtles in aquaculture. Am J Vet Res. 1975;36(08):1221-4. Epub 1975/08/01. PubMed PMID: 168798.

119. Haines H. A herpesvirus disease of green sea turtles in aquaculture. Marine F~sheries Review. 1978:33-7.

120. Curry SS, Brown DR, Gaskin JM, Jacobson ER, Ehrhart LM, Blahak S, et al. Persistent infectivity of a disease-associated herpesvirus in green turtles after exposure to seawater. Journal of Wildlife Diseases. 2000;36(4):792-7.

121. Coberley SS, Herbst LH, Brown DR, Ehrhart LM, Bagley DA, Schaf SA, et al. Detection of antibodies to a disease-associated herpesvirus of the green turtle, Chelonia mydas. Journal of clinical microbiology. 2001;39(10):3572-7.

122. Stacy BA, Wellehan JFX, Foley AM, Coberley SS, Herbst LH, Manire CA, et al. Two herpesviruses associated with disease in wild Atlantic loggerhead sea turtles (Caretta caretta). Veterinary Microbiology. 2008;126(1–3):63-73. doi: http://dx.doi.org/10.1016/j.vetmic.2007.07.002.

123. Manire CA, Stacy BA, Kinsel MJ, Daniel HT, Anderson ET, Wellehan JF. Proliferative dermatitis in a loggerhead turtle, Caretta caretta, and a green turtle, Chelonia mydas, associated with novel papillomaviruses. Veterinary microbiology. 2008;130(3):227-37.

124. Herbst LH, Lenz J, Van Doorslaer K, Chen Z, Stacy BA, Wellehan Jr JFX, et al. Genomic characterization of two novel reptilian papillomaviruses, Chelonia mydas papillomavirus 1 and Caretta caretta papillomavirus 1. Virology. 2009;383(1):131-5. doi: http://dx.doi.org/10.1016/j.virol.2008.09.022.

125. Mashkour N, Maclaine A, Burgess GW, Ariel E. Discovery of an Australian Chelonia mydas papillomavirus via green turtle primary cell culture and qPCR. J Virol Methods. 2018. Epub 2018/04/10. doi: 10.1016/j.jviromet.2018.04.004. PubMed PMID: 29630942.

126. Casey RN, Quackenbush SL, Work TM, Balazs GH, Bowser PR, Casey JW. Evidence for retrovirus infections in green turtles Chelonia mydas from the Hawaiian islands. Diseases of Aquatic Organisms. 1997;31(1):1-7. doi: 10.3354/dao031001.

127. Ng TFF, Manire C, Borrowman K, Langer T, Ehrhart L, Breitbart M. Discovery of a Novel Single-Stranded DNA Virus from a Sea Turtle Fibropapilloma by Using Viral Metagenomics. Journal of Virology. 2009;83(6):2500-9. doi: 10.1128/JVI.01946-08. PubMed PMID: PMC2648252.

128. Gilman E, Zollett E, Beverly S, Nakano H, Davis K, Shiode D, et al. Reducing sea turtle by‐catch in pelagic longline fisheries. Fish and Fisheries. 2006;7(1):2-23.

129. Work PA, Sapp AL, Scott DW, Dodd MG. Influence of small vessel operation and propulsion system on loggerhead sea turtle injuries. Journal of Experimental Marine Biology and Ecology. 2010;393(1):168-75. doi: http://dx.doi.org/10.1016/j.jembe.2010.07.019.

130. Crane O. Marine Turtle Health Assessment and Aquarium Suitability: Uzi Island, Zanzibar. 2013.

131. Deem SL, Dierenfeld ES, Sounguet GP, Alleman AR, Cray C, Poppenga RH, et al. Blood values in free-ranging nesting leatherback sea turtles (Dermochelys coriacea) on the coast of the Republic of Gabon. Journal of Zoo and Wildlife Medicine. 2006;37(4):464-71.

132. Mettee N. Wound Management/V.A.C. The Wider Caribbean Sea Turtle Conservation Network (WIDECAST) Technical Report No. 16: 2014.

133. Mettee N. Buoyancy Disorders. The Wider Caribbean Sea Turtle Conservation Network (WIDECAST) Technical Report No. 16: 2014.

134. Schmitt T, Leger JS, Munns S, Adams L, editors. Pulmonary function testing in healthy and positively buoyant olive ridely sea turtles (*Lepidochelys olivacea*). IAAAM 36th Annaul Conference Proceedings; 2005; Seward, Alaska.

135. Lutz PL, Musick JA. The biology of sea turtles, Volume I: CRC press; 1996.

136. Fauquier DA, Flewelling LJ, Maucher J, Manire CA, Socha V, Kinsel MJ, et al. Brevetoxin in blood, biological fluids, and tissues of sea turtles naturally exposed to Karenia brevis blooms in central west Florida. Journal of Zoo and Wildlife Medicine. 2013;44(2):364-75.

137. Hawkes LA, Broderick AC, Godfrey MH, Godley BJ. Climate change and marine turtles. Endangered Species Research. 2009;7(2):137-54.

138. Davenport J. Temperature and the life-history strategies of sea turtles. Journal of thermal biology. 1997;22(6):479-88.

139. Shaver DJ, Tissot PE, Streich MM, Walker JS, Rubio C, Amos AF, et al. Hypothermic stunning of green sea turtles in a western Gulf of Mexico foraging habitat. PLOS ONE. 2017;12(3):e0173920. doi: 10.1371/journal.pone.0173920.

140. Carr A. Impact of nondegradable marine debris on the ecology and survival outlook of sea turtles. Marine Pollution Bulletin. 1987;18(6):352-6.

141. Adimey NM, Hudak CA, Powell JR, Bassos-Hull K, Foley A, Farmer NA, et al. Fishery gear interactions from stranded bottlenose dolphins, Florida manatees and sea turtles in Florida, U.S.A. Marine Pollution Bulletin. 2014;81(1):103-15. doi: http://dx.doi.org/10.1016/j.marpolbul.2014.02.008.

142. Mendonca; V, Abi-Aoun B. The sea turtle Chelonia mydas population at Ras Al Hadd Nature Reserve: Turtle nesting density and strands, and turtle predator abundance on Ras Al Jinz beaches – June 2009 Records Ras Al Jinz Scientific and Visitor Centre, 2009.

143. Bezjian M, Wellehan JF, Jr., Walsh MT, Anderson E, Jacobson E. Management of wounds in a loggerhead sea turtle (Caretta caretta) caused by traumatic bycatch injury from the spines of a spotted eagle ray (Aetobatus narinari). J Zoo Wildl Med. 2014;45(2):428-32. Epub 2014/07/09. doi: 10.1638/2013-0178r.1. PubMed PMID: 25000714.

144. Brodie J, Ariel E, Thomas C, O’Brien D, Berry K. Links between water quality and marine turtle health. TropWATER - Tropical Water & Aquatic Ecosystem Research: 2014.

145. Camedda A, Marra S, Matiddi M, Massaro G, Coppa S, Perilli A, et al. Interaction between loggerhead sea turtles (Caretta caretta) and marine litter in Sardinia (Western Mediterranean Sea). Marine environmental research. 2014;100:25-32.

146. Deem SL, Norton TM, Mitchell M, Segars A, Alleman AR, Cray C, et al. Comparison of blood values in foraging, nesting, and stranded loggerhead turtles (Caretta caretta) along the coast of Georgia, USA. Journal of Wildlife Diseases. 2009;45(1):41-56.

147. Guzmán MM, Rodríguez SJ. Accumulation and tissue distribution of metals and other elements in sea turtles from all over the world. Turtles: Anatomy, Ecology and Conservation: Nova Science Publishers, Inc.; 2011. p. 1-48.

148. Jacobson ER, Homer BL, Stacy BA, Greiner EC, Szabo NJ, Chrisman CL, et al. Neurological disease in wild loggerhead sea turtles Caretta caretta. Dis Aquat Organ. 2006;70(1-2):139-54. Epub 2006/08/01. doi: 10.3354/dao070139. PubMed PMID: 16875401.

149. Flint M, Patterson-Kane JC, Limpus CJ, Mills PC. Health surveillance of stranded green turtles in Southern Queensland, Australia (2006–2009): an epidemiological analysis of causes of disease and mortality. EcoHealth. 2010;7(1):135-45.

150. Manire CA, Charles AM, Eric TA, Lynne B, Deborah AF. DEHYDRATION AS AN EFFECTIVE TREATMENT FOR BREVETOXICOSIS IN LOGGERHEAD SEA TURTLES (CARETTA CARETTA). Journal of zoo and wildlife medicine. 2013;44(2):447-52. doi: 10.1638/2012-0163R.1.

151. Sloan K. Barnacle growth as an indicator of the onset and duration of the clinical symptoms of debilitated turtle syndrome affecting loggerhead (Caretta caretta) sea turtles: College of Charleston; 2011.

152. Norton TM. Debilitated Sea Turtle Clinical Management. WIDECAST Technical Report No. 16: 2014.

153. Fernández I, Retamal MA, Mansilla M, Yáñez F, Campos V, Smith C, et al. Analysis of epibiont data in relation with the Debilitated Turtle Syndrome of sea turtles in Chelonia mydas and Lepidochelys olivacea from Concepción coast, Chile/Análisis de los datos de epibiontes en relación con el Síndrome de Debilitamiento de Tortugas marinas en Lepidochelys olivacea y Chelonia mydas de la costa de Concepción, Chile. Latin American Journal of Aquatic Research. 2015;43(5):1024-9. doi: http://dx.doi.org/10.3856/vol43-issue5-fulltext-23. PubMed PMID: 1761238821.
