# Supplementary material for: Disease risk analysis in sea turtles: A baseline study to inform conservation efforts
Source: PLoS One. 2020 Oct 23;15(10):e0230760. doi: 10.1371/journal.pone.0230760 (PMC7584443; doi:10.1371/journal.pone.0230760)
Supplement: Attachment — Submitted filename: Response to reviewers.docx [file pone.0230760.s003.docx]

Response to reviewers’ comments for “Disease Risk Analysis in sea turtles: A baseline study to inform conservation efforts”

By Narges Mashkour et al.

**Reviewer one**

| Reviewer’s Comments | Responses to reviewer | Change in the text | Pages in manuscript |
| --- | --- | --- | --- |
| The largest issue I have with the manuscript is the lack of a dedicated section on the threats posed to sea turtles by global warming. | This section was primarily a part of the manuscript which was removed later on, as it was a controversial topic as a direct disease causing factor.  We have included the subsection in results and explained how debatable it can be. | Global temperature is increasing and may directly influence species or the pattern of their lives (123, 124). Migratory species are more vulnerable to temperature change, especially if they are forced to undertake large distance migration for breeding or changing seasonal habitats (123). Climate change can also alter pathogen survival, transmission patterns, ecological balances, vector and host susceptibility (124, 125). Such adverse impacts have been seen in FP prevalence and the development of clinical disease (76).  Sea turtles exhibit temperature dependent sex determination and global warming is proved to exacerbate female biased offspring in these species. The sex ratio of 90% females is reported in some nests which is reducing the chance of reproduction in a population (126). At birth, hurricanes and other storm events inundate the nests and lead to hatching failure (86, 127). After birth, climate change can threaten their habitat when severe weather events disturb the sea grass meadow, lead to coral bleaching and influence the water quality (95). Climate change is adversely affecting sea turtle populations, however to directly link global warming and the health of sea turtles and to describe it as a causative agent for diseases, further investigations are required.  In 2010, two independent "Expert Opinion Surveys" were done to rank the threats for sea turtles. The results were inconsistent; one group ranked the global climate change and pathogens the least important threat for sea turtles (128) while the other group gave the highest rank to global warming (122). Experts' opinion around these impacts is filled with uncertainty, which is reflected in the very different ranking of climate change in the two publications. The authors believed the differences in ranking came from the recent identification or emergence of these hazards which also emphasise the need for further investigations on this issue (128). | Section 3.1.3. “Global Warming and diseases”  is added to the results |
| The authors highlight the difficulty of studying marine species such as  sea turtles and their diseases. The authors should comment briefly on the potential of current and future advances to better facilitate such research, for example comment on the potential of emerging eDNA technologies | A section is added to introduction mentioning advanced technologies such as eDNA and satellite telemetry. The benefits and challenges of using both techniques is discussed. | Advanced biodiversity monitoring techniques for sea turtles conservation is gaining popularity including alternative and efficient techniques such as environmental DNA (8, 9). Screening eDNA is considered to be a non-invasive way to assess the population dynamics of sea turtles (10), along with investigating the causation and progression of diseases such as fibropapillomatosis (FP) (11). Satellite telemetry is another advanced tool successfully used in sea turtle population assessment and marine parks management (12).  However such technologies are still evolving and face challenges that make them not globally accessible; eDNA for instance, requires finances, advanced laboratory equipment and skilled operators to analyse and interpret the results, otherwise it may not provide accurate information (13). The satellite deployment sites are biased towards for example North America rather than Africa due to the cost of developing, maintaining and utilising these techniques (14). | Introduction line number 16 to 26 |
| Trauma and injuries’ section: Please cite literature more extensively, boat strikes are  a well-established issue for sea turtles (and other marine species), while a welcome inclusion, relying on a single anecdotal source is not sufficient for this section. | This topic is mentioned in different sections of the manuscript.  More citations were added. | Air breathing marine species are at risk of vessel collision and sea turtles are no exception (12). The data clearly indicates that the vessel strike injuries occur in both protected marine environments and remote areas (99).  As examples, during 2000–2014 in Florida, between 1,326 and 4,334 sea turtles mostly green and loggerhead were killed after a boat strike incident (100). In the French Caribbean, boat strikes appear to occur at a higher rate in the past few years (personal interview with the veterinarian in Saint Barthelemy/Saint Martin FWI). The incidences are lethal in the majority of cases with a very low survival rate (1/10 sea turtles survived after intensive veterinary care at Saint Barthelemy/Saint Martin FWI in 2019). In Galapagos Marine Reserve, incidences mostly happen in commercial tourism areas and there is no data on survival rates (99).  To diminish this threat different laws and regulations have been imposed (101) and to reduce the deadly impacts several conservation approaches were introduced such as educating vessel operators on how to spot sea turtles, establishing go‐slow zones or no‐entry areas and increasing the use of propulsion systems to avoid exposed propellers (12, 100). Mortality caused by fishing gear is also troubling sea turtle populations around the world. The data from a 5-year study (2013-2017) in the Mid-Atlantic and Northeast has reported the alarming mortality rates caused by different fishing gears: trawl, 48%; gillnet, 73%; dredge, 40%; vertical, 55% and fish trap, 57% (102). As explained in Canary Islands, Spain, fishing is associated with net entanglement and hook and monofilament line ingestion which lead to death (52). Implementing changes in fishing strategies, operations and technologies have shown to reduce the mortality rates associated with fishing worldwide (103, 104). An example is using turtle exclusion devices (TED) to be able to release sea turtles from trawls (103). | 3.1.2.1. Trauma and injuries |
| 3.1.2.3. Gastrointestinal disorders’ section: The particular susceptibility to post-hatchling life  stages to plastic ingestion should be highlighted, for example see White et al. 2018, as should the propensity for ingested macroplastic fragments to result in cellular microplastic presence, with currently unknown health consequences | The issue is expanded. | Gastrointestinal disorders are one of the main concerns for sea turtles in rehabilitation centres (43). Gastrointestinal obstruction by debris such as plastic is a clear risk for turtles (110, 111). Macroplastic ingestion is ranked at the same level as other anthropogenic pressures such as by-catch (Duncan et al, 2019). The risk of plastic ingestion has been documented for decades and has affected different age groups of sea turtles (White et al, 2018, Stamper et al, 2009, Torrent et al, 2002). As a result, in post hatchlings and juvenile turtles, gastrointestinal impaction or injury directly affect the health and reduced nutrient intake or abnormal buoyancy indirectly impair the fitness (White et al, 2018). Generally, the risk of macroplastic ingestion is documented more than microplastics and in some cases is reported to have higher impacts. Micronising plastic can accumulate in coastal sediments and in ocean beds but the consequences for sea turtles is still unclear (Duncan et al, 2019). | 3.1.2.3. Gastrointestinal disorders |
| Table 3. Gram positive bacteria ‘Unfortunately there was not enough time to go through this  list.’. While I appreciate that there may have been limited time at the workshops, could the  authors arrange a virtual meeting (e.g. Zoom) or online poll of the relevant participants to go  through and rank this list? I would recommend this, and the inclusion of the top 3 ranked  gram positive bacteria in this table. | The group in the second workshop reviewed both groups and decided not to separate bacteria by Gram categories.  We also had two microbiologists who actively work with sea turtles, review the categories, so we don’t feel it would add to the manuscript to conduct a further workshop on this topic. | NA | NA |
| Also Table 3, Cladosporium: ‘hatching failure, infections in captivity’. ‘Hatching’ should  have a capital ‘H’ to match the formatting of the other rows. | Done! | Hatching | Table 3, Cladosporium |
| Section 3.3.1: ‘These interactions can positively or negatively impact the stakeholders.’  Depending on your intended meaning I think this should read:  ‘… positively or negatively impact sea turtles’,  or ‘… positively or negatively impact sea turtles and those trying to protect them’,  or ‘… positively or negatively impact sea turtles and human stakeholders of the marine  environment’, | Thanks for pointing this out | Changed to: …positively or negatively impact sea turtles and those trying to protect them | 3.3.1. Sea turtle and One Health consideration in the literature |
| Table 5, Section C. Although occurring after the time of the workshops, it would be worth noting here the extremely detrimental effect the current covid19 pandemic is having on the viability of ecotourism models. There is already a lot of anecdotal evidence in the media that the decline in travel is severely affecting programmes that are primarily supported through ecotourism. | Thanks for the suggestion.  The explanation is added to the table in form of a footnote. | *The global covid19 pandemic has had an adverse effect on ecotourism. Conservation organisations that depend on the funds from ecotourism are at risk of closure or financial crisis. Overfishing and poaching have been reported during lock-down. Conservationists are urged to rethink how to achieve their goals post-pandemic (141). | Table 5, footnote |
| Sections 3.4.2.1. and 3.4.2.2. might be better suited to the appendix/supplemental material.  Although present as local case studies, they do not add as much to the overall global picture, therefore I would suggest moving them. | This section was written in response to the co-authors concern about management units and to localise the assessed risk.  The co-authors believed that some risks can be better mitigated if they are reviewed on a small scale.  And this practice is an example for the readers to hopefully be able to follow these steps. |  |  |
| Appendix 7 page 53: ‘Surgery is done in some areas but after rehabilitation and release,  tumour regrowth may happen and low probability of success is reported in Southeastern  parts of USA’  asked to change for: ‘Surgery is conducted in some areas, in some cases after  rehabilitation and release, tumour regrowth may occur and there is a varying probability of  rehabilitation success reported in Southeastern parts of the USA’. | Thanks for the comprehensive explanation. | Changed to  Surgery is conducted in some areas, in some cases after rehabilitation and release, tumour regrowth may occur and there is a varying probability of rehabilitation success reported in Southeastern parts of the USA. | Appendix 7 page 63 |
| Appendix 7 page 54: ‘The only sea turtle herpes virus successfully isolated in cell culture’.  Do you mean that in this study when samples were used in cell culture that this was the only  herpesvirus isolated, or that this is the only sea turtle herpesvirus ever isolated from cell  culture? Please clarify. If the latter, ChHV5 has recently also been cultured using a cell  culture model. | The text is modified to clarify the meaning. | The first sea turtle herpesvirus isolated in cell culture. | Appendix 7 page 64 |

**Reviewer two**

| Reviewer’s Comments | Responses to reviewer | Change in the text | Pages in manuscript |
| --- | --- | --- | --- |
| The “materials and method” section is too long and some sentences sound as results or discussions. | This manuscript is method based and if it’s going to be used as a guideline the materials and methods need to be comprehensive.  However this section was reviewed by some co-authors and a section was moved to results. | This section has been moved to results under gastrointestinal disorder:  Generally, the risk of macroplastic ingestion is higher than that for microplastics. Macroplastic ingestion is a concern for conservation, ranked at the same level as other anthropogenic pressures such as by-catch (112). | From section 2.5.1. to 3.1.2.3. Gastrointestinal disorders |
| There is neither a “discussion” section nor a “conclusion” section, that is replaced with a summary. May be a discussion of the results and of the process that generate the DRA should be provided. | The “summary section” is a discussion about the process of DRA as suggested in the comment.  The results of this DRA are discussed in each section and also assessed as parts of DRA process.  To avoid confusion, the section is titled discussion now. | The section is titled discussion. | Section 4. Discussion |
