# Supplementary material for: Disease risk analysis in sea turtles: A baseline study to inform conservation efforts
Source: PLoS One. 2020 Oct 23;15(10):e0230760. doi: 10.1371/journal.pone.0230760 (PMC7584443; doi:10.1371/journal.pone.0230760)
Supplement: Attachment — Submitted filename: Reviewer comments v1.5.pdf [file pone.0230760.s002.pdf]

The authors present comprehensive materials to facilitate future sea turtle DRAs, in addition they provide an overview of current global threats to sea turtles, derived from an extensive literature search and from the inclusion of expert opinion obtained at local and international workshops. Due to its value for establishing a baseline, and encouraging future systematic disease risk analyses I believe that upon the completion of some minor revision the manuscript would be highly suitable for publication in PLOS ONE.

Minor revisions:

The largest issue I have with the manuscript is the lack of a dedicated section on the threats posed to sea turtles by global warming. While mentioned in passing throughout some sections this threat has the potential to rapidly become the most damaging to sea turtle health and disease states with far reaching consequences. Indeed this was highlighted as a top non-infectious risk by participants of both workshops (Table 3). I strongly recommend the addition of a dedicated sub-section in the Results section dealing explicitly with the myriad of global warming associated problems sea turtles are facing. Such threats relating to the ongoing climate emergency include (but are not limited to), effects on sex ratios, increased incidence of disease, loss of habitat (nesting [sea level rise] and oceanic [altered water temperatures]), destabilised climatic conditions leading to increased frequency of sever events (storms, hurricanes etc. and resulting increase in nest inundations etc), coral bleaching (reefs being a key habitat), effects on sea grass meadows and ocean acidification (effects on prey).

Introduction section. The authors highlight the difficulty of studying marine species such as sea turtles and their diseases. The authors should comment briefly on the potential of current and future advances to better facilitate such research, for example comment on the potential of emerging eDNA technologies to aid somewhat with the detection of sea turtles and their pathogens/parasites in the marine environment[1-4].

'3.1.2.1. Trauma and injuries' section: Please cite literature more extensively, boat strikes are a well-established issue for sea turtles (and other marine species), while a welcome inclusion, relying on a single anecdotal source is not sufficient for this section. For example extensive and detailed vessel-strike stranding information is present in Foley et al. 2015 [5]. Literature on this area is relative extensive, a few more examples being Denkinger et al. 2013, Shimada et al. 2017 and Orós et al. 2005 [6-8]. Foley et al 2015 also collate shark bite, fishing line and hook information, topics which also require more literature to be cited in this section.

'3.1.2.3. Gastrointestinal disorders' section: The particular susceptibility to post-hatchling life stages to plastic ingestion should be highlighted, for example see White et al. 2018[9], as

should the propensity for ingested macroplastic fragments to result in cellular microplastic presence, with currently unknown health consequences.

Table 3. Gram positive bacteria '*Unfortunately there was not enough time to go through this list.*'. While I appreciate that there may have been limited time at the workshops, could the authors arrange a virtual meeting (e.g. Zoom) or online poll of the relevant participants to go through and rank this list? I would recommend this, and the inclusion of the top 3 ranked gram positive bacteria in this table.

Also Table 3, Cladosporium: '*hatching failure, infections in captivity*'. '*Hatching'* should have a capital 'H' to match the formatting of the other rows.

Section 3.3.1: 'These interactions can positively or negatively impact the stakeholders.' Depending on your intended meaning I think this should read:

'… positively or negatively impact sea turtles',

or '… positively or negatively impact sea turtles and those trying to protect them',

or '… positively or negatively impact sea turtles and human stakeholders of the marine environment',

Table 5, Section C. Although occurring after the time of the workshops, it would be worth noting here the extremely detrimental effect the current covid19 pandemic is having on the viability of ecotourism models. There is already a lot of anecdotal evidence in the media that the decline in travel is severely affecting programmes that are primarily supported through ecotourism.

Sections 3.4.2.1. and 3.4.2.2. might be better suited to the appendix/supplemental material. Although present as local case studies, they do not add as much to the overall global picture, therefore I would suggest moving them.

Appendix 7 page 53: '*Surgery is done in some areas but after rehabilitation and release, tumour regrowth may happen* ***and low probability of success*** *is reported in Southeastern parts of USA*'

This sentence is misleading. Probability of success is proportional to severity of disease. For example, in Florida 44% of FP tumor score 1 sea turtles are successfully rehabilitated and

released (Stage 2: 22%, and Stage 3: 6.4%)[10]. Many turtles stranding with FP have co-morbidities (emaciation, entanglement, boat-strikes etc.), and these figures do not differentiate between when non-successful rehabilitation of FP-afflicted turtles was due to FP or these other non-FP factors. Furthermore, the ability to treat and rehabilitate FP-afflicted sea turtles continues to be refined, for example the introduction of laser surgery, and a number of studies demonstrating effectiveness of a variety of human anti-cancer therapeutics for the treatment of FP. The figures reported above cover the period from 2006-2016 and do not necessarily take account of the latest advances or their effect on rehabilitation success.

Therefore, this sentence should be altered to more accurately reflect the success or otherwise of rehab, please change to: '*Surgery is conducted in some areas, in some cases after rehabilitation and release, tumour regrowth may occur and there is a varying probability of rehabilitation success reported in Southeastern parts of the USA*'.

While comprehensive assessment is lacking, sea turtles previously rehabilitated for FP have subsequently been identified nesting (many years later). Conversely some rehabilitated turtles have re-stranded with FP regrowth.

Appendix 7 page 54: '*The only sea turtle herpes virus successfully isolated in cell culture*'. Do you mean that in this study when samples were used in cell culture that this was the only herpesvirus isolated, or that this is the only sea turtle herpesvirus ever isolated from cell culture? Please clarify. If the latter, ChHV5 has recently also been cultured using a cell culture model[11].

References:

1 Harper, K. J. *et al.* Finding Crush: environmental DNA analysis as a tool for tracking the green sea turtle Chelonia mydas in a marine estuary. *Frontiers in Marine Science* (2020).

2 Yetsko, K. *et al.* Mutational, transcriptional and viral shedding dynamics of the marine turtle fibropapillomatosis tumor epizootic. *bioRxiv*, 2020.2002.2004.932632, doi:10.1101/2020.02.04.932632 (2020).

3 Thomsen, P. F. & Willerslev, E. Environmental DNA – An emerging tool in conservation for monitoring past and present biodiversity. *Biol. Conserv.* **183**, 4-18, doi:http://dx.doi.org/10.1016/j.biocon.2014.11.019 (2015).

4 Bohmann, K. *et al.* Environmental DNA for wildlife biology and biodiversity monitoring. *Trends Ecol. Evol.* **29**, 358-367, doi:10.1016/j.tree.2014.04.003 (2014).

5 Foley, A. M. *et al.* Distributions, relative abundances, and mortality factors of sea turtles in Florida during 1980–2014 as determined from strandings. *Fish and Wildlife Research Institute, Jacksonville Field Laboratory, Jacksonville, Florida, USA* (2015).

6 Denkinger, J. *et al.* Are boat strikes a threat to sea turtles in the Galapagos Marine Reserve? *Ocean Coast. Manage.* **80**, 29-35, doi:https://doi.org/10.1016/j.ocecoaman.2013.03.005 (2013).

7 Shimada, T., Limpus, C., Jones, R. & Hamann, M. Aligning habitat use with management zoning to reduce vessel strike of sea turtles. *Ocean Coast. Manage.* **142**, 163-172, doi:https://doi.org/10.1016/j.ocecoaman.2017.03.028 (2017).

8 Oros, J., Torrent, A., Calabuig, P. & Deniz, S. Diseases and causes of mortality among sea turtles stranded in the Canary Islands, Spain (1998–2001). *Dis. Aquat. Org.* **63**, 13-24 (2005).

9 White, E. M. *et al.* Ingested Micronizing Plastic Particle Compositions and Size Distributions within Stranded Post-Hatchling Sea Turtles. *Environ. Sci. Technol.* **52**, 10307-10316, doi:10.1021/acs.est.8b02776 (2018).

10 Stacy, B. A. *et al.* Report of the Technical Expert Workshop: Developing Recommendations for Field Response, Captive Management, and Rehabilitation of Sea Turtles with Fibropapillomatosis. doi:https://doi.org/10.25923/adg2-g771 (2019).

11 Work, T. M., Dagenais, J., Weatherby, T. M., Balazs, G. H. & Ackermann, M. In-vitro replication of Chelonid herpesvirus 5 in organotypic skin cultures from Hawaiian green turtles (Chelonia mydas). *J. Virol.* **91**, e00404-00417 doi:10.1128/jvi.00404-17 (2017).
